# Ferritin Conjugates With Multiple Clickable Amino Acids Encoded by C-Terminal Engineered Pyrrolysyl-tRNA Synthetase

**DOI:** 10.3389/fchem.2021.779976

**Published:** 2021-11-25

**Authors:** Yi-Hui Wang, Mu-Lung Jian, Pei-Jung Chen, Jo-Chu Tsou, Le P. Truong, Yane-Shih Wang

**Affiliations:** ^1^ Institute of Biological Chemistry, Academia Sinica, Taipei, Taiwan; ^2^ Institute of Biochemical Sciences, College of Life Science, National Taiwan University, Taipei, Taiwan

**Keywords:** pyrrolysyl-tRNA synthetase, ferritin-drug conjugates, strain-promoted azide-alkyne cycloaddition, p-Azido-l-phenylalanine, amber suppression efficiency, drug delivery

## Abstract

This study reports the application of expanding genetic codes in developing protein cage-based delivery systems. The evolved Methanosarcina mazei pyrrolysyl-tRNA synthetase (PylRS)•tRNA^Pyl^ pairs derived from directed evolution are examined to probe their recognition for para-substituted phenylalanine analogs. The evolved MmPylRS, AzFRS, harboring a wide range of substrates, is further engineered at the C-terminal region into another variant, AzFRS-MS. AzFRS-MS shows suppression of the elevated sfGFP protein amount up to 10 TAG stop codons when charging p-azido-l-phenylalanine (AzF, **4**), which allows the occurrence of click chemistry. Since protein nanocages used as drug delivery systems that encompass multiple drugs through a site-specific loading approach remain largely unexplored, as a proof of concept, the application of AzFRS-MS for the site-specific incorporation of AzF on human heavy chain ferritin (Ftn) is developed. The Ftn-**4** conjugate is shown to be able to load multiple fluorescence dyes or a therapeutic agent, doxorubicin (Dox), through the strain-promoted azide-alkyne cycloaddition (SPAAC) click reaction. Aiming to selectively target Her2^+^ breast cancer cells, Ftn-**4**-DOX conjugates fused with a HER2 receptor recognition peptide, anti-Her2/neu peptide (AHNP), is developed and demonstrated to be able to deliver Dox into the cell and to prolong the drug release. This work presents another application of evolved MmPylRS systems, whose potential in developing a variety of protein conjugates is noteworthy.

## Introduction

Protein cages consist of a self-assembled protein hollow shell, such as viral capsids, virus-like particles, chaperonins, and human ferritin heavy chain (Ftn) (Aumiller et al., 2018). These biological supermolecules formed by multiple copies of monomer possess symmetric three-dimensional structures with remarkable stability. Protein cages are widely used as delivery platforms in bio-nanotechnology and material science. In the field of drug delivery, protein cages are employed to prolong the half-life of drugs, exert enhanced permeability and retention effect, reduce nonspecific uptake by introducing targeting ligand, and enhance drug solubility or endocytosis effect (Zhang et al., 2021). For instance, Ftn is applied to the delivery of enzymes (Tetter and Hilvert, 2017; Uchida et al., 2018) and chemotherapy drugs (Ahn et al., 2018; Song et al., 2021).

**FIGURE 1:**
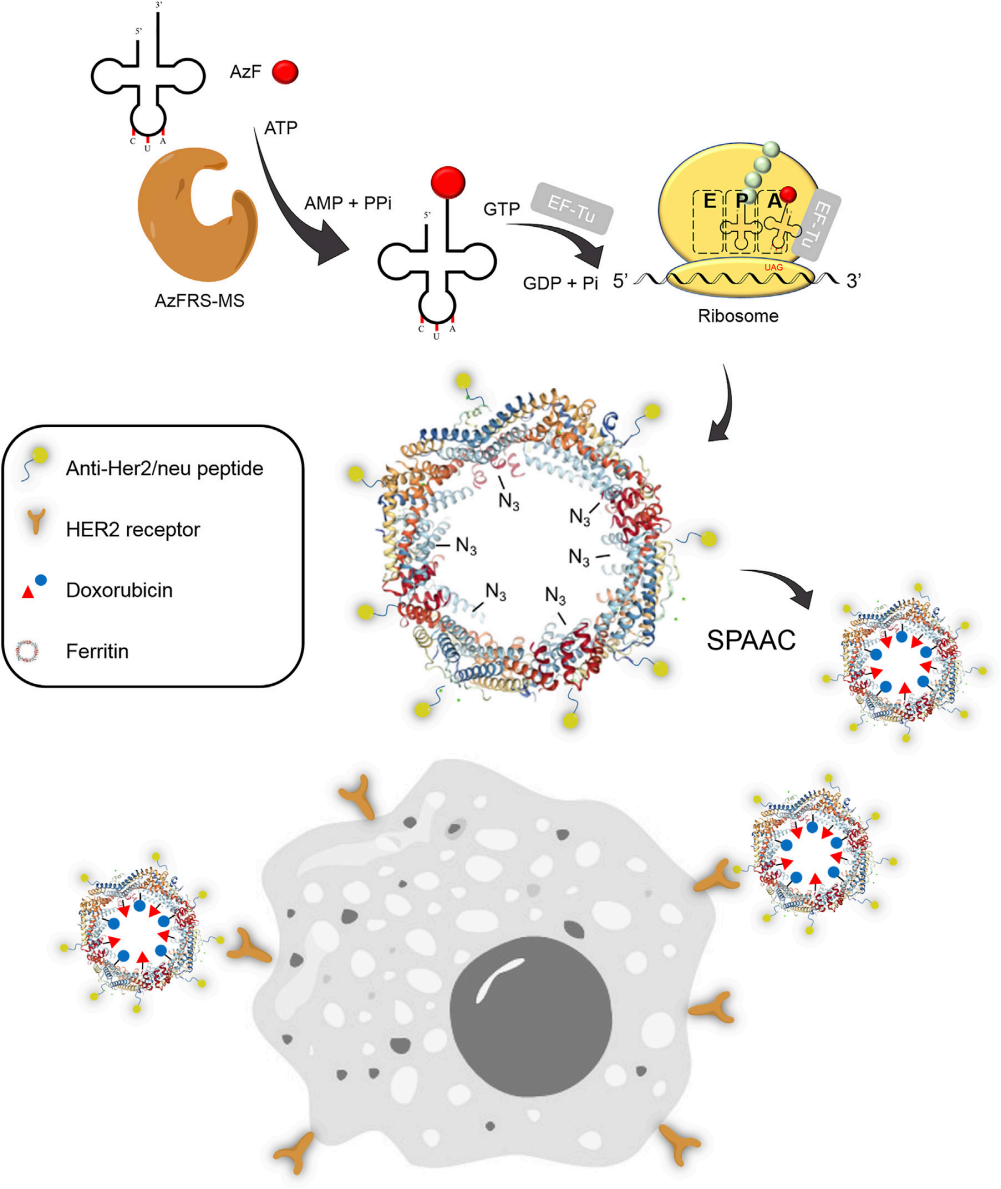
Synthesis of the Ftn–Dox conjugate and the targeting of Her2^+^ breast cancer cells. The Ftn–Dox conjugate is synthesized through the expanding genetic code approach and the SPAAC reaction. The engineered AzFRS-MS•tRNA^Pyl^ pair is used to incorporate AzF (**4**, red filled circle) into Ftn. The installed azido group is shown as −N_3_ on the Ftn nanocage. The Ftn–Dox conjugate with the installed anti-Her2/neu peptide (AHNP) facilitates the targeting of breast cancer cells, BT474, in this study. SPAAC, strain-promoted alkyne-azide cycloaddition.

Ftn was firstly discovered and isolated from horse spleen. It is found to be highly conserved across all kingdoms of life and serves as an iron transporter in biological systems. Ftn consists of 24 subunits that can self-assemble into a spherical structure with an octahedral symmetry and an outer diameter of around 12 nm. The N-terminal region of Ftn is pointed outward, whereas the C-terminal E-helix of Ftn is pointed inward (Figures 1, Figures 4A) (Theil, 1987). For recognition purposes, the embedded targeting motifs are usually designed to be genetically fused to the N-terminus of Ftn, which allows 24 targeting motifs exposed on the Ftn surface to specifically recognize the target expressed on cells. The modified Ftn has been applied in cancer therapies (Song et al., 2021; Zhang et al., 2021).

In addition, Ftn maintains the assembled nanocage structure at the temperature up to 80°C and remains stable at the pH range of 4–12. Ftn can reversibly assemble the cage from disassembled monomers at neutral pH (Kim et al., 2011). Due to its physicochemical robustness, Ftn is an ideal candidate to be covalently modified into a *de novo* drug delivery platform.

**SCHEME 1:**
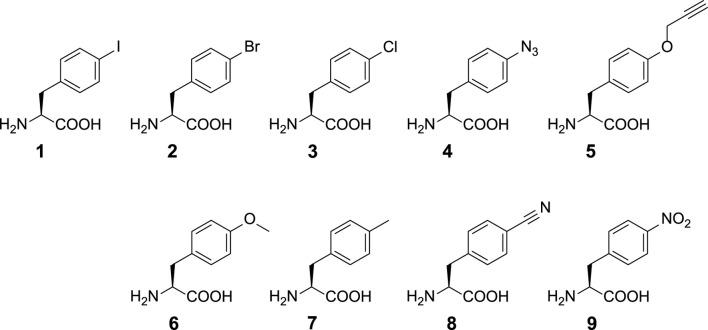
Chemical structures of ncAAs used in this study. ncAA **1**: *p*-iodo-l-phenylalanine (IF); **2**: *p*-bromo-l-phenylalanine (BrF); **3**: *p*-chloro-l-phenylalanine (ClF); **4**: *p*-azido-l-phenylalanine (AzF); **5**: *p*-propargyl-l-phenylalanine (PrF); **6**: *p*-methoxy-l-phenylalanine (MeOF); **7**: *p*-methyl-l-phenylalanine (MeF); **8**: *p*-cyano-l-phenylalanine (CNF); **9**: *p*-nitro-l-phenylalanine (NO_2_F).

The approach installing versatile and bioorthogonal functional groups by expanding genetic codes has become a practical method for the preparation of protein conjugates and is able to incorporate diverse bioorthogonal click chemistry pairs on proteins (Liu and Schultz, 2010). For instance, some potent *in vivo* bioorthogonal chemical reactions achieved through the expansion of genetic codes are copper(I)-catalyzed azide-alkyne cycloaddition (CuAAC), strain-promoted alkyne-azide cycloaddition (SPAAC), and strain-promoted inverse electron-demand Diels–Alder cycloaddition (Lang and Chin, 2014). The bioorthogonal pyrrolysyl-tRNA synthetase (PylRS)•tRNA^Pyl^ pairs from archaea *Methanosarcina mazei (Mm)* (Yanagisawa et al., 2006; Kavran et al., 2007; Chen et al., 2009) and *Methanosarcina barkeri* (Gautier et al., 2010), as well as eubacteria *Desulfitobacterium hafniense (Dh)* (Lee et al., 2008; Nozawa et al., 2009), are widely engineered to incorporate non-canonical amino acids (ncAAs), mainly for lysine (Cigler et al., 2017; Xuan et al., 2017; Abdelkader et al., 2021), phenylalanine (Phe, e.g., ncAAs **1–9**, Scheme 1) (Wang et al., 2011; Wang et al., 2012; Wang et al., 2013), tryptophan (Englert et al., 2015; Jiang et al., 2020b), histidine (Xiao et al., 2014; Sharma et al., 2016), cysteine (Nguyen et al., 2014), homoarginine (Mukai et al., 2015), and aspartic acid analogs (Xuan et al., 2018) into proteins *in vivo*. Therefore, clickable ncAAs such as azide, propargyl, *bi*-cyclooctyne, *trans*-cyclooctene, or tetrazine functional groups can be developed through these pairs (Wan et al., 2014; Tsai et al., 2015). The CuAAC and SPAAC reactions on proteins are demonstrated using genetically encoded *p*-azido-l-phenylalanine (AzF, **4**, Scheme 1) produced through amber TAG stop codon suppression by evolved *Methanococcus jannaschii* TyrRS•*Mj*tRNA^Tyr^ pair in the *Escherichia coli* system (Chin et al., 2002). A semi-rationally designed *Dh*PylRS•*Dh*tRNA^Pyl^ pair is also found to be able to charge **4** in *E. coli* (Fladischer et al., 2019). In addition, evolved *E. coli* TyrRS (E2AziRS)•*Ec*tRNA^Tyr^ (Sakamoto et al., 2002; Coin et al., 2013) is applied to incorporate **4** onto proteins in mammalian cells. Among these pairs, the *Mm*PylRS•tRNA^Pyl^ pair is shown to offer proper bioorthogonality spanning from prokaryotes to eukaryotes. Structural and engineering studies that contributed in the understanding of interactions among the N-terminal domain (NTD) (Suzuki et al., 2017; Sharma et al., 2018), the active site, and the linker region connecting the NTD and the active site (Suzuki et al., 2017; Jiang et al., 2020a) of *Mm*PylRS have been conducted. *Mm*PylRS harbors a large and dynamic active site; thus, the directed evolution of *Mm*PylRS leads to the development of mutants with higher suppression efficiency and the expansion of substrate ranges. Nevertheless, the *Mm*PylRS•tRNA^Pyl^ pair has not evolved for charging **4** in *E. coli* and mammalian cells yet.

In this article, the application of rationally designed *Mm*PylRS variants in the preparation of a fluorophore- or drug-loaded Ftn delivery system is reported. The *Mm*PylRS variants with mutations on the C-terminal domain (CTD) that enhances amber suppression for multiple **4** incorporations on Ftn are developed to enable the clickable chemistry, i.e., SPAAC, for the conjugation of fluorophores or drugs. Using the engineered *Mm*PylRS•tRNA^Pyl^ pair, a **4**-incorporated Ftn delivery system is designed to target a breast cancer overly expressed human epidermal growth factor receptor 2 (HER2-positive; Her2^+^), which is more malignant and has a higher incidence as well as mortality rate among all breast cancer subtypes (Piccart-Gebhart et al., 2005; Romond et al., 2005). The N-terminus of this Ftn delivery system is fused with anti-Her2/neu peptide (AHNP) (Park et al., 2000), a peptide of 12 amino acids derived from trastuzumab which specifically binds to the HER2 receptor with high affinity. The chemotherapeutic agent, doxorubicin (Dox), whose mode of action occurs through its intercalation into dsDNA in the nucleus, is conjugated on this Ftn delivery system *via* the clickable SPAAC reaction. The successful results herein demonstrate the feasibility of similar designs for the development of chemical moiety-loaded protein cage systems and also provide another example for the versatile use of expanded genetic codes.

## Materials and methods

### General strains and plasmid construction

The ncAAs **1–9** were purchased from Chem-Impex Inc. (Wood Dale, IL, USA). Polymerase chain reaction (PCR) was performed using the KOD Hot Start Polymerase kit (Merck, Bedford, MA, USA). The oligonucleotide synthesis and DNA sequencing service were provided by Genomics Inc. (Taipei, Taiwan). The *sfGFP-10ams* gene, amber mutations at 10 phenylalanine positions F8/F27/F46/F71/F100/F114/F130/F145/F165/F223, and human heavy chain ferritin *Ftn* gene synthesis with *E. coli* codon optimization were performed by Integrated DNA Technologies (IDT) Inc. The primer sequences in this study are listed in the Supplementary Materials. The construction of plasmids pET-sfGFP-27am, pET-sfGFP-2∼8ams, and sfGFP-10ams followed the general cloning protocols. The *sfGFP-27am* gene was generated using the *sfGFP* gene amplified by PCR from pET-pylT-sfGFP (Jiang et al., 2020a) and then prepared by overlapping PCR with two fragments (fragment 1: sfGFP-NdeI-F and 27am-R; fragment 2: 27am-F and sfGFP-SacI-R). The overlapped *sfGFP-27am* gene products and pET-pylT plasmid were double digested with restriction enzymes NdeI and SacI and then ligated using T4 DNA ligase. Similarly, pET-pylT plasmids harboring sfGFP with multiple TAG amber stop codons were constructed with multiple fragments by overlapping PCR. Briefly, the *sfGFP-2ams* gene with F27/F46 amber mutations was synthesized using three fragments: fragment 1, sfGFP-NdeI-F and 27am-R; fragment 2, 27am-F and 46am-R; and fragment 3, 46am-F and sfGFP-SacI-R. The *sfGFP-3ams* gene, with amber mutations at F8/F71/F100, was prepared using the following three fragments by overlapping the PCR method: fragment 1, NdeI-F8am-F and 71am-R; fragment 2, 71am-F and 100am-R; and fragment 3, 100am-F and sfGFP-SacI-R. The *sfGFP-4ams* gene with F27/F84/F100/F165 amber mutations was amplified by overlapping PCR using the following five fragments: fragment 1, sfGFP-NdeI-F and 27am-R; fragment 2, 27am-F and 84am-R; fragment 3, 84am-F and 100am-R; fragment 4, 100am-F and 165am-R; and fragment 5, 165am-F and sfGFP-SacI-R. The *sfGFP-5ams* gene with F27/F84/F100/F114/F165 amber mutations was prepared by two fragments (fragment 1: sfGFP-NdeI-F and 114am-R; and fragment 2: 114am-F and sfGFP-SacI-R), using pET-sfGFP-4ams as the template. The *sfGFP-6ams* gene with F27/F84/F100/F114/F165/F223 amber mutations was prepared by two fragments (fragment 1: sfGFP-NdeI-F and 223am-R; and fragment 2: 223am-F and sfGFP-SacI-R), using pET-sfGFP-5ams as the template. The *sfGFP-7ams* gene with the additional F8 amber mutation on the *sfGFP-6ams* gene was prepared by primers NdeI-F8am-F and sfGFP-SacI-R, using pET-sfGFP-6ams as the template. The *sfGFP-8ams* gene with S2 amber mutation on the *sfGFP-7ams* gene was prepared by primers NdeI-F2am-F and sfGFP-SacI-R, using pET-sfGFP-7ams as the template. The amber codon positions for sfGFP variants are listed in Supplementary Table S1.

For the construction of the pET-Ftn plasmid, the synthesized *Ftn* gene was inserted into the pET-pylT plasmid using the same approach as to that for the preparation of pET-sfGFP-27am. For the construction of pET-Ftn-F81am, pET-Ftn-K143am, and pET-Ftn-2ams, respectively, the *Ftn-F81am*, *Ftn-K143am*, and *Ftn-2ams* genes were synthesized from wild-type *Ftn* genes with F81 and/or K143 amber mutations by overlapping PCR. Briefly, the *Ftn-F81am* gene was generated by overlapping two fragments, fragment 1: Ftn-NdeI-F and F81am-R and fragment 2: F81am-F and Ftn-SacI-R, using the template pET-Ftn. The *Ftn-K143am* and *Ftn-2ams* genes were synthesized by the same approach using two fragments, fragment 1: Ftn-NdeI-F and K143am-R; and fragment 2: K143am-F and Ftn-SacI-R, using templates pET-Ftn and pET-Ftn-F81am, respectively. The plasmids pET-A-Ftn and pET-Ftn-A were also constructed by introducing genes encoding the N- or C-terminal AHNP peptide and linker (GGGGS)_3_ to the *Ftn* gene to generate *A-Ftn* and *Ftn-A* genes. The *A-Ftn* and *Ftn-A* genes were synthesized with two fragments using pET-Ftn as the template: fragment 1 (N-AHNP-F and N-AHNP-R for *A-Ftn* construction; whereas C-ANHP-F and C-ANHP-R for *Ftn-A*) and fragment 2 (3XG4S-Ftn-F and 3XG4S-Ftn-R for *A-Ftn*, whereas Ftn-3XG4S-C-ANHP-F and Ftn-3XG4S-C-ANHP-R for *Ftn-A*). The plasmids pET-A-Ftn-F81am, pET-A-Ftn-K143am, and pET-A-Ftn-2ams were constructed utilizing the same approach, with the same primers for the construction of the *A-Ftn* gene and using pET-Ftn-F81am, pET-Ftn-K143am, and pET-Ftn-2ams as the templates, respectively.

*Mm*PylRS variants, IFRSs, evolved from directed evolution (Wang et al., 2011) which charge *p*-iodo-l-phenylalanine (IF, **1**), were generated with designated mutations on the active site (Table 1). Briefly, for the construction of the plasmid of pCDF-IFRS1, the *IFRS1* gene product was generated by overlapping PCR with three fragments using the template pCDF-PylRS (Jiang et al., 2020b), fragment 1: EcoR1-F and IFRS1-MLS-R; fragment 2: IFRS1-MLS-F and IFRS1-SM-R; and fragment 3: IFRS1-SM-F and BamHI-R. The overlapped *IFRS1* gene product and pET-pylT plasmid were then double digested with restriction enzymes EcoRI and BamHI and then ligated using T4 DNA ligase. pCDF-IFRS2 was constructed by modifying the *IFRS1* gene of pCDF-IFRS1, and the overlapping PCR product was generated with two fragments, fragment 1: EcoR1-F and IFRS2-TA-R and fragment 2: IFRS1-TA-F and BamHI-R. For the construction of pCDF-AzFRS, the *AzFRS* gene product was generated with three fragments using pCDF-PylRS as the template, fragment 1: EcoRI-F and AzFRS-AM-R; fragment 2: AzFRS-AM-F and AzFRS-L-R; and fragment 3: AzFRS-L-F and BamHI-R. Using pCDF-AzFRS as the DNA template, different combinations of C-terminal mutations (listed in the Supplementary Materials), K431M, D433G, and A441S, were introduced through the designed primers to generate *AzFRS-M*, *AzFRS-G*, *AzFRS-S*, *AzFRS-MG*, *AzFRS-MS*, *AzFRS-GS*, and *AzFRSc* genes (Table 1).

**TABLE 1:** PylRS variants employed in this study.

PylRS	L305	Y306	L309	N346	C348	W417	K431	D433	A441
IFRS1^a^	M	L	S	S	M				
IFRS2^a^		T	A	S	M				
AzFRS^a^				A	M	L			
AzFRSc				A	M	L	M	G	S
AzFRS-M				A	M	L	M		
AzFRS-G				A	M	L		G	
AzFRS-S				A	M	L			S
AzFRS-MG				A	M	L	M	G	
AzFRS-MS^b^				A	M	L	M		S
AzFRS-GS				A	M	L		G	S

a These three PylRS variants are directed evolved from plasmid library selection against ncAA **1**.

b This variant is used for both ncAA-encoded sfGFP and ferritin protein production.

### Expression and purification of ncAA-encoded sfGFP and Ftn proteins

To produce ncAA-encoded sfGFP proteins, the pET-sfGFP-27am and pCDF-AzFRS-MS co-transformed into *E. coli* BL21 (DE3), sequentially. After the transformation, the cell suspension was spread onto the agar plate containing ampicillin (Amp) (100 μg/ml) and streptomycin (Sm) (100 μg/ml). A single colony was chosen from the plate and then cultured in 1 ml Luria–Bertani (LB) medium overnight. The cultured cells were then transferred to 50 ml fresh LB medium and incubated at 37°C until OD_595_ reached 0.6–0.8. Followed by centrifugation (10 min, 6,000 rpm, 4°C), the medium is changed to GMML medium. Protein expression was induced with the supplement of 1 mM isopropyl β-D-1-thiogalactopyranoside (IPTG) and ncAA and incubated at 37°C for another 12 h. Cells were harvested, resuspended with lysis buffer (1× phosphate-buffered saline [PBS], pH 7.4), and then sonicated to lyse cells. Followed by centrifugation (60 min, 20,000 rpm, 4°C), the supernatant was collected. As sfGFP was designed with a 6× His tag, the supernatant collected was incubated with 0.5 ml Ni^2+^-NTA resin (Roche, Basel, Switzerland) for protein purification. Five milliliters of lysis buffer and 2.5 ml of washing buffer (1× PBS, 5 mM imidazole, pH 7.4) were used to wash out nonspecific proteins on the resin. The target protein was eluted from the resin by 2.5 ml of elution buffer (1× PBS, 200 mM imidazole, pH 7.4). The buffer of eluted fraction was changed to 1× PBS with Amicon Ultra-15 Centrifugal Filter Units (MWCO 10 kDa). For Ftn proteins, further purification by anion exchange column HiTrap Q HP (GE Healthcare, Chicago, IL, USA) was conducted with NaCl gradient elution buffer (50 mM Tris, 50 mM to 1 M NaCl, pH 8.0) and size exclusion chromatography (SEC) column HiLoad 10/300 Superdex 200 pg (GE Healthcare) with PBS buffer. Purified sfGFP and Ftn were analyzed by 12% SDS-PAGE with Instant Blue (Marvelgent Biosciences, Canton, MA, USA) staining. The Ftn assembling status was analyzed by native gel, 4%–15% Mini-PROTEAN^®^ TGX™ Precast Protein Gel (Bio-Rad, Hercules, CA, USA) with a native protein marker (Thermo Fisher Scientific, Waltham, MA, USA) in TG buffer (25 mM Tris–HCl, 192 mM glycine, pH 8.5) under 80 V for 6 h at 4°C. The gel was stained by Instant Blue for 15 min and destained by H_2_O.

### Western blot analysis

Whole cells were collected and lysed at 100°C with SDS loading dye for 15 min and then subjected to 12% SDS-PAGE analysis. The gels were stained with Instant Blue to visualize the target proteins with the expected molecular weight around 28 kDa corresponding to the protein size of sfGFP. The suppression efficiency of amber codons in producing sfGFP with the C-terminal His tag was visualized by Western blotting against the anti-6X His tag antibody. The Western blots were performed using Trans-Blot Turbo System (Bio-Rad) and RTA transfer kit. The antibodies used for immunoblotting were Anti-His (SignalChem, Richmond, Canada; H99-61M-100) and HRP-conjugated secondary antibody (Cell Signaling Technology, Danvers, MA, USA; 7076P2). After SDS-PAGE analysis, the gel was immersed in the transfer buffer and then blotted onto the polyvinylidene fluoride (PVDF) membrane (25 V/1.3 A, 10 min). After the completion of the transfer process, the PVDF membrane was washed with PBST buffer for 5 min thrice. Next, the membrane was blocked with 5% skim milk for 1 h at room temperature. The membrane was then washed with PBST buffer for 5 min thrice (washing step). A primary antibody (1:1,000 dilution) was added and then incubated with the membrane for 1 h at room temperature following the washing step. Subsequently, an HRP-conjugated secondary antibody (1:5,000 dilution) was added and the membrane was incubated for another 1 h at room temperature. The membrane was then washed. Finally, the WesternBright ECL HRP substrate (Advansta, San Jose, CA, USA; K-12045-D50) was mixed and spread onto the membrane to visualize the band signals using ChemiDoc Imaging Systems (Bio-Rad) in bioluminescence detection mode.

### ESI-MS characterization of sfGFP and Ftn proteins

The purified protein was diluted with 50% acetonitrile and 1% formic acid. An aliquot corresponding to one pmol of the pure protein was injected *via* an ESI source (Waters LockSpray Exact Mass Ionization Source) with a syringe pump (Harvard Apparatus, Holliston, MA, USA), and a flow rate of 5 μl/min was held throughout the analysis. The mass of intact proteins was determined using a Waters SYNAPT G2 HDMS mass spectrometer (Waters, Milford, MA, USA). The acquired spectra were deconvoluted to the single-charge state using the MaxEnt1 algorithm of the MassLynx 4.1 software (Waters).

### Determination for the suppression efficiencies of MmPylRS variants

To understand the suppression of *Mm*PylRS variants, ncAA **1**–**9** screening assay was performed. The plasmids pET-sfGFP-27am and pCDF-PylRS were co-transformed into *E. coli* BL21 (DE3), sequentially. The cell suspension was spread onto the agar plate containing Amp (100 μg/ml) and Sm (100 μg/ml). The plate was incubated at 37°C overnight. Ten colonies were selected and then inoculated and cultured in 3 ml of LB medium at 37°C overnight. Five hundred microliters of cells was transferred to 25 ml of fresh LB medium and incubated at 37°C until OD_595_ reached 0.6–0.8. The cells were harvested, washed twice with M9 salts, and suspended in M9 medium (M9 salts, 1% glycerol, 2 mM MgSO_4_ and 0.1 mM CaCl_2_) supplemented with 1 mM IPTG. Aliquots of 50 μl suspended cells were loaded in a 384-well plate supplemented with **1–9** ncAAs (1 mM) in designated wells. Cells were incubated in the plate reader (BioTek, Winooski, VT, USA) at 37°C for 12 h, with continuous monitoring of fluorescence emission intensity (*λ*_ex_/*λ*_em_ = 535/595 nm) as well as OD_595_. The control experiments used to measure background signals were performed for wells without adding IPTG and ncAAs, or wells adding IPTG only. The control signal (without adding ncAAs but IPTG) was subtracted from the fluorescence emission intensity of sfGFP obtained and then divided by OD_595_ to generate the relative fluorescence intensity.

### Synthesis of Ftn-Dye and A-Ftn–DOX conjugates

To synthesize Ftn-dye conjugates, purified Ftn-81-**4**, Ftn-143-**4**, Ftn-2am-**4**, A-Ftn-81-**4**, A-Ftn-143-**4**, and A-Ftn-2am-**4** proteins were conjugated to fluorescence dyes or Dox by the SPAAC reaction. The DBCO-Cy3 and DBCO-Cy5 (Sigma-Aldrich, St. Louis, MO, USA) were dissolved in 100% DMSO to the final concentration of 1 mM. The Ftn-81-**4** protein (20 μl, 20 μM) in PBS buffer at pH 7.4 was mixed with 50 μl of DBCO-Cy3 and/or DBCO-Cy5 (80 μM, 1:5 M ratio) in the buffer of 90% PBS and 10% DMSO, and then the volume was adjusted to 500 μl. Reaction was incubated at 37°C, 300 rpm for 16 h. A-Ftn-81-TAMRA conjugates were synthesized by mixing A-Ftn-81-**4** and DBCO-PEG4-TAMRA (Sigma-Aldrich) under the same condition. The Ftn-dye conjugates were further purified using the SEC column (HiLoad 10/300 Superdex 200 pg, GE Healthcare) and then subjected to SDS-PAGE, native PAGE, gel fluorescence imaging, and ESI-MS analysis. A-Ftn-143-Dox was synthesized by coupling A-Ftn-143-**4** and dibenzocyclooctyne–PEG4–doxorubicin (DBCO–PEG4–DOX) using the same method. DBCO–PEG4–DOX was synthesized by NHS-amine coupling chemistry. DBCO–PEG4–NHS (Sigma-Aldrich) (50 μl, 1.5 mM, 30% DMSO in PBS) and doxorubicin hydrochloride (Dox, TCI) (50 μl, 2 mM, 30% DMSO in PBS) were mixed and reacted at 25°C for 4 h. The chemical reaction progress was monitored by normal-phase thin-layer chromatography.

### Dynamic light scattering, transmission electron microscopy, and fluorescence spectroscopic analysis of Ftn variants

To analyze the particle size by dynamic light scattering (DLS), Ftn variants were diluted to the final concentration of 0.5 mg/ml and the aggregates were removed from the solution by centrifuge. The protein samples were loading into the designated 1 ml disposable cuvettes, and the particle size of protein was measured by Malvern Zetasizer Nano ZS. Measurements were repeated three times, and the primary size distribution by intensity value was determined.

To prepare Ftn samples for transmission electron microscope (TEM) analysis, 50 μg/ml of protein was dissolved in 50 mM Tris buffer at pH 8.0. After the support film grid (formvar/carbon 400 mesh copper) discharged with 25 mA for 30 s by Emitech K100X Glow, 5 μl of protein was loaded onto the grid and incubated for 60 s. The sample was gently drained from the grid by filter paper. Distilled deionized water and 1% uranyl acetate (UA) were alternatively applied onto the grid, incubated for 30 s, and drained by filter paper three times for background cleaning. Images of Ftn proteins were collected on FEG-TEM, FEI Tecnai G2 TF20 Super TWIN. Microscopy was operated at an accelerating voltage of 120 kV.

The fluorescence emission spectra were determined by Fluorolog-3 (Jobin Yvon, Edison, NJ, USA). Proteins were diluted to 50 μg/ml in PBS at pH 7.4 and loaded into the designated 10-mm quartz cuvettes. Ftn-143-Cy3, Ftn-143-Cy5, and Ftn-143-Cy3/Cy5 were excited at 550 nm. The emission spectra were recorded from 560 to 700 nm with 1 nm per point scanning resolution.

### *In vitro* BT474 cell targeting study, confocal microscopy imaging, and cytotoxicity assay

Human breast carcinoma (BT-474) cells (BCRC, Taiwan) were cultured in 45% high-glucose DMEM (Corning, Tewksbury, MA, USA) and 45% low-glucose DMEM (Corning, USA) supplemented with 10% fetal bovine serum (FBS, HyClone, Logan, UT, USA) and 1% penicillin-streptomycin (Gibco, Grand Island, NY, USA) at 37°C with 5% CO_2_ in a humid atmosphere in petri dishes (Falcon). Cells were ready for the detachment when the confluency reached above 60%. BT-474 cells were rinsed with PBS twice and then trypsinized with 0.25% trypsin–EDTA (Gibco, USA). An appropriate volume of trypsin-EDTA was added into petri dishes. After gently shaking, cells were incubated with trypsin–EDTA at 37°C for 1 min. The detached cells were collected by pipetting over the cell layer with fresh complete medium. Twenty microliters of suspended cells was taken and mixed with trypan blue for cell density measurement. Cell density was determined by an automated cell counter (Invitrogen, Carlsbad, CA, USA). One-third of cells was added into a new petri dish and diluted to the appropriate volume with fresh complete medium. Poly-d-lysine (Sigma-Aldrich) coated coverslips were used for BT-474 cell attachment. Coverslips were incubated with 50 μg/ml poly-d-lysine for 2 h. Subsequently, the solution was removed and then the coverslips were rinsed with PBS and sterile water three times, alternatively. The coated coverslips were allowed to dry for at least 2 h before introducing cells and medium. Suspended cells (1 × 10^5^) were seeded on 18-mm coverslips within a 12-well plate and incubated for 24 h, allowing cells to attach on coverslips.

After cells were attached on coverslips, the culture medium was replaced by fresh DMEM containing Dox, fluorescence molecules, A-Ftn-dye, or A-Ftn-drug conjugates. The cells were incubated at 37°C for the designated time and washed twice with PBS solution. The cells were fixed on coverslips by incubating with 4% para-formaldehyde (PFA) (Sigma-Aldrich) for 10 min. PFA was removed and the coverslips rinsed twice with PBS. After equilibrating the coverslips with PBS, the cells were ready to be stained. The nucleus of cells was stained by DAPI (GeneCopoeia, Rockville, MD, USA). An appropriate volume of 300 nM DAPI was added into a culture plate with coverslips and incubated for 5–10 min. Then, DAPI was removed and the coverslips alternatively rinsed with PBS and sterile H_2_O three times. The coated coverslips were allowed to dry for at least 5 min before mounting. The cells were mounted onto a slide with one drop or 10 μl of mounting medium, ProLong Gold Antifade Mountant (Invitrogen). The cells were observed under the corresponding channel by confocal microscopy (Leica SP5).

The cytotoxicity of the A-Ftn–DOX conjugate, A-Ftn-**4,** and free Dox, respectively, against Her2^+^ human breast cancer cells, BT-474, was evaluated by 3-(4,5-dimethylthiazol-2-yl)-2,5-diphenyltetrazolium bromide (MTT, Thermo) assay. As the confluency of BT-474 reached above 60%, the cells were detached and 1 × 10^4^ cells were seeded onto a 96-well transparent flat plate per well with 100 μl of fresh medium. The cells were further incubated for 24 h to allow the cell to attach onto the plate. Subsequently, the free Dox, A-Ftn-**4**, and A-Ftn–DOX conjugates were added into designated wells with the final concentration ranging from 0 to 5 μM. After the incubation for 72 h, the medium was removed and replaced by 100 μl of 1.2 mM MTT in PBS per well, following the 4-h incubation. The MTT solution was carefully removed, and DMSO was added into wells to dissolve the purple formazan crystals. The absorption at 540 and 570 nm was measured by Synergy HT plate reader (Biotech).

## Results

### Efficient MmPylRS variants for p-azido-L-phenylalanine (4) incorporation at multiple sites on proteins

**FIGURE 2:**
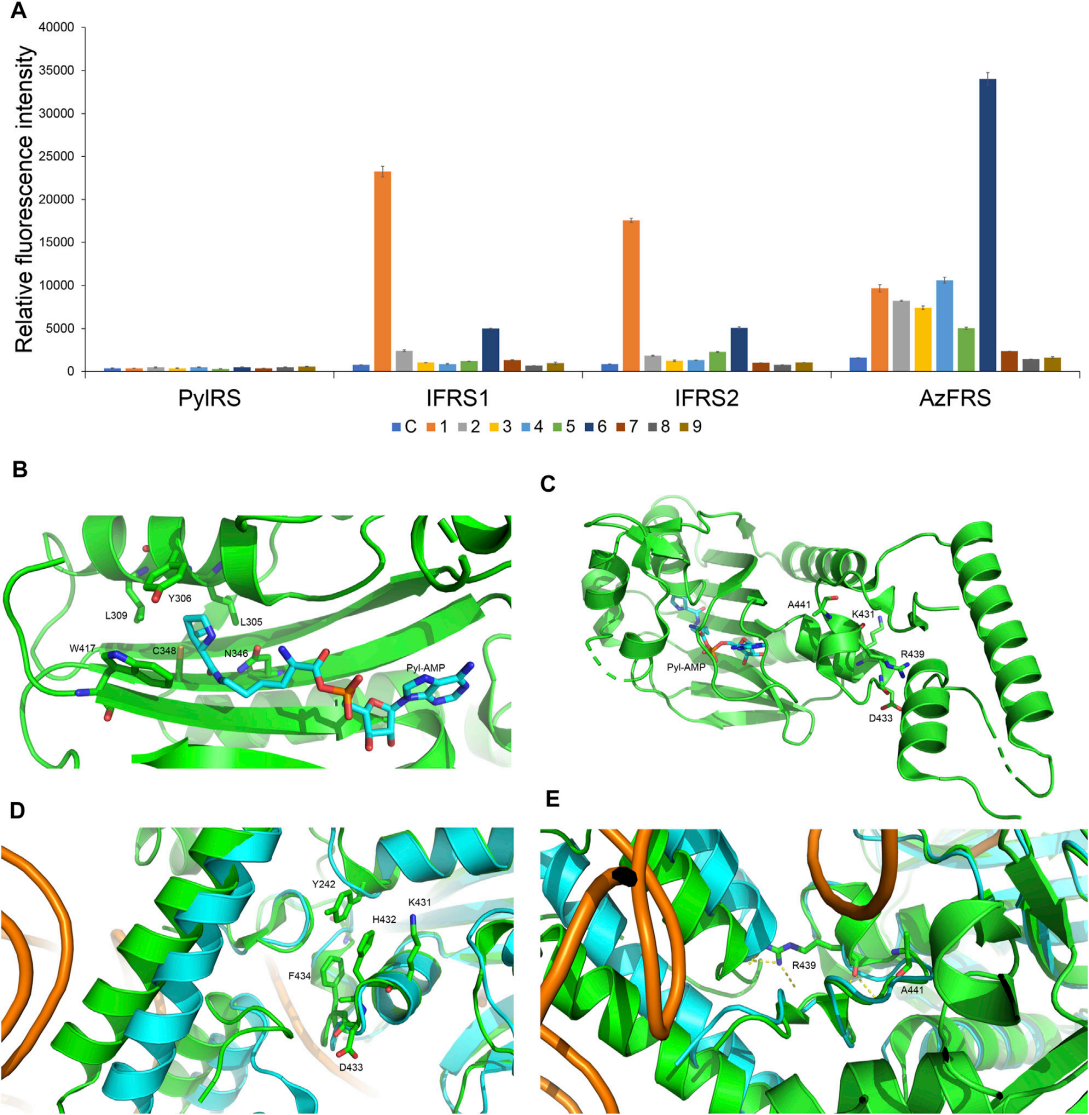
Substrate specificity screening and C-terminal designs of evolved *Mm*PylRS. **(A)** The incorporation efficiencies of PylRS variants, IFRS1, IFRS2, and AzFRS (Table 1), are determined by fluorescence emission intensities obtained from *sfGFP-27am* gene products. Proteins are expressed after being supplemented with 1 mM ncAA and IPTG in GMML medium at 37°C for 12 h. Cells are excited at 485 nm, and the fluorescence emission intensities are detected at 535 nm. The cell density is monitored by the UV absorbance at 595 nm. C denotes the control experiment that cells were supplemented with 1 mM IPTG only; **1–9** denote the experiments supplemented with 1 mM IPTG and ncAAs **1–9** (Scheme 1). The background signal obtained from cells without adding IPTG is subtracted from the signal obtained from each group. Error bars represent the standard deviation of sfGFP production from three repeated experiments. **(B)** The active site of *Mm*PylRS. **(C)** The structure of *Mm*PylRS CTD in the presence of Pyl-AMP substrate obtained from the X-ray co-crystal structure (PDB code: 2Q7H). The AMP-Pyl and six mutated sites from directed evolution are shown in stick mode. The designed mutation sites, K431, D433, and A441, in this study are labeled. The residue R439 is also labeled to indicate the spatial arrangement toward the first helix (position 191–201). **(D)** and **(E)** The structure of *Mm*PylRS CTD (green) and its superimposition with the *Dh*PylRS CTD (cyan)/tRNA^Pyl^ (orange) complex. The structures are illustrated based on two PDB entries 2Q7H and 2ZNI. The amino acids proposed to interact with the mutated site are labeled. The yellow dotted line indicates the hydrogen bonding.

To incorporate AzF (**4**), the evolved PylRS•tRNA^Pyl^ pair (Wang et al., 2011) is chosen from the *Mm*PylRS plasmid library (Wang et al., 2010) to be fine-tuned in charging ncAA **1**. The wild-type *Mm*PylRS and three IFRS variants, IFRS1, IFRS2, and AzFRS (Table 1), are selected to explore the substrate recognition against nine *para*-substituted phenylalanine analogs, **1**–**9** (Scheme 1; Figure 2A), respectively. The mutations at the active site of these three *Mm*PylRS variants cover two conserved mutation sites at N346 and C348, together with randomly distributed mutations at other sites (Table 1; Figure 2B). Both the pCDF vector containing the mutated PylRS gene and the pET vector containing tRNA^Pyl^ and the *sfGFP-27am* gene are co-introduced into *E. coli*. The read-through of the amber codon in response to ncAAs **1**–**9** (Scheme 1), respectively, can be indicated through the fluorescence emission resulting from the *sfGFP-27am* gene product. The screening results obtained from both IFRS1 and IFRS2 give the preference in charging IF (**1**) and MeOF (**6**) (Figure 2A), indicating that both IFRS1 and IFRS2 show high substrate specificity. All AzFRS variants share W417L/N346A/C348M mutation sites (Table 1). The screening results of AzFRS variants give the high preference when charging **6**, whereas mild preferences are observed when charging **1**–**5**. AzF (**4**) and *p*-propargyl-l-phenylalanine (PrF, **5**), from which versatile functional groups can be derived for click reactions such as SPAAC and CuAAC, show noticeable signal enhancement when being charged by AzFRS.

To enhance the activity of *Mm*PylRS variants, the engineering for the second sphere of the active site or the terminal sequence of *Mm*PylRS is conducted. Although the second sphere or global sequence engineering (Suzuki et al., 2017; Sharma et al., 2018; Jiang et al., 2020a) of *Mm*PylRS has been studied, the effects of *Mm*PylRS CTD mutations on amber suppression efficiency remain unclear. Based on the available x-ray crystal structure, mutations at the CTD for rationally designed AzFRS have been introduced and studied (Figure 2C). Among the evolved *Mm*PylRS variants, HarRS, which charges homoarginine with K431M/D433G/G444E mutations at HarRS CTD (Mukai et al., 2015), is found to be able to enhance the activity and selectivity for homoarginine incorporation. Likewise, in this study, to develop the hypothesized remodeled pi–pi interactions among Y242, H432, and F434 positions, K431M/D433G double mutations are chosen for the CTD mutation sites due to their strong hydrophobicity (Figures 2C,D). In addition, the A441S mutation is performed to affect the adjacent hydrogen bond network built by the main chain of the R439 residue and to indirectly participate in the hydrogen bond network between the side chain of R439 and the neighboring alpha helix (Figure 2C, positions 191–201), whose spatial movement is found in the *Dh*PylRS CTD/tRNA^Pyl^ (cyan) co-crystal structure (Figures 2D,E). Those mobile helical regions (green and cyan helices on the left side of Figure 2E) are interfaced with the tRNA D-loop region based on the superimposed structures of the *Dh*PylRS CTD/tRNA^Pyl^ co-crystal (cyan, Figure 2E) and the *Mm*PylRS CTD (green, Figure 2E).

**FIGURE 3:**
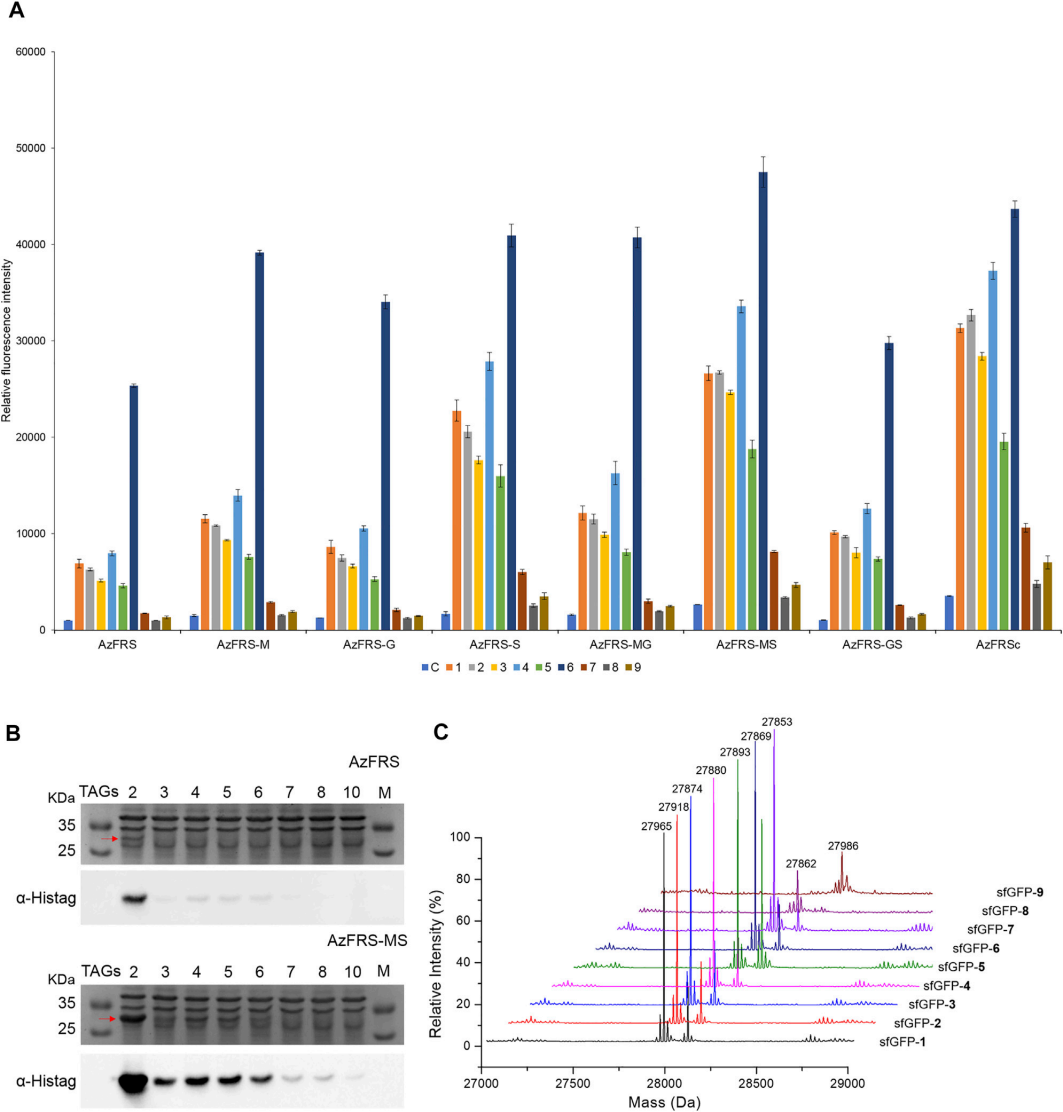
sfGFP production, amber suppression efficiency, and mass characterization obtained from AzFRS variants. **(A)** The incorporation efficiencies of AzFRS variants (Table 1) are determined by the fluorescence emission intensities obtained from *sfGFP-27am* gene products, respectively. Proteins are expressed after supplemented with 1 mM ncAA and IPTG in GMML medium at 37°C for 12 h. Cells are excited at 485 nm, and the fluorescence emission intensities are recorded at 535 nm. The cell density is monitored through the UV absorbance at 595 nm. C denotes the control experiment that cells supplemented with 1 mM IPTG only; **1–9** denote the experiments supplemented with 1 mM IPTG and ncAAs **1–9** (Scheme 1). The background signal obtained from cells without adding IPTG is subtracted from the signal obtained from each group. Error bars represent the standard deviation of sfGFP production from three repeated experiments. **(B)** Tandem amber codon suppression efficiency analysis for sfGFP production by AzFRS•tRNA^Pyl^ and AzFRS-MS•tRNA^Pyl^ pairs. Amber suppression of *sfGFP-2ams∼8ams* and *sfGFP-10ams* genes, containing suppression of 2∼10 TAG stop codons, produces full-length sfGFP proteins with the incorporation of multiple **4**. The sfGFP proteins are overexpressed in *E. coli* BL21 (DE3) coding AzFRS•tRNA^Pyl^ or AzFRS-MS•tRNA^Pyl^ pair supplemented with 1 mM IPTG and 1 mM **4** in GMML medium at 37°C for 12 h. The whole-cell lysate is analyzed by SDS-PAGE and Western blotting against the anti-Histag antibody (indicated as α-Histag). The red arrow indicates the sfGFP protein band on the SDS-PAGE. The SDS-PAGE and Western blotting analysis are shown in Supplementary Figures S1, S2; **(C)** Mass determination of *sfGFP-27am* gene products coupled with ncAAs **1–9** by ESI-MS. The full-length sfGFP-**1–9** proteins are overexpressed by the AzFRS-MS•tRNA^Pyl^ pair in *E. coli* BL21 (DE3) supplemented with 1 mM IPTG and 1 mM ncAAs **1**–**9** in GMML medium at 37°C for 12 h. The calculated and observed molecular masses of sfGFP-**1–9** are found in Table 2. The detailed electrospray and deconvoluted mass spectrum are shown in Supplementary Figures S3–S11.

Upon the introduction of three mutations, K431M/D433G/A441S, on *Mm*PylRS to evolve into the AzFRS variant, another three variants with a single mutation at the AzFRS CTD are prepared to examine its effect: AzFRS-M, AzFRS-G, and AzFRS-S (Table 1). Through monitoring the fluorescence emitted from the *sfGFP-27am* gene product, it is noted that, compared with AzFRS, AzFRS-M and AzFRS-G give the 1.4- and 1.6-fold enhancement in the fluorescence emission in charging MeOF (**6**), respectively, and give the 1.6- and 1.3-fold enhancement in the fluorescence emission in charging AzF (**4**), respectively (Figure 3A). Compared with AzFRS, AzFRS-S yields 2- to 4.3-fold enhancement in the fluorescence emission when charging **1–6**, which shows the improved activity toward those ncAAs. To examine the likelihood whether a combination of those mutations can further elevate the activity of AzFRS, AzFRS variants with double mutations, i.e., AzFRS-MG, AZFRS-MS, and AzFRS-GS, as well as the AzFRS variant with triple mutations, i.e., AzFRSc, are prepared and tested with the same approach. Interestingly, D433G mutation does not seem to contribute to the enhanced activity. The AzFRS-MG variant merely shows a neutral effect, whereas the AzFRS-GS variant even shows a negative effect. Relative to AzFRS, AzFRS-MS and AzFRSc variants yield a 4-fold increase in the fluorescence emission when charging **4**. Based on these results, AzFRS-MS is chosen in charging **4** in the subsequent studies.

The amber suppression efficiencies of AzFRS, AzFRSc, and AzFRS-MS are evaluated through the generation of *sfGFP-2ams*, *sfGFP-3ams*, *sfGFP-4ams*, *sfGFP-5ams*, *sfGFP-6ams* ***,*** *sfGFP-7ams*, *sfGFP-8ams*, and *sfGFP-10ams* gene products (Amiram et al., 2015) (Figure 3B; Supplementary Figures S1, S2). With the similar endogenous protein background, the whole-cell SDS-PAGE and Western blotting analysis of full-length sfGFP indicate an improvement in the suppression efficiency. AzFRS and AzFRS-MS can overly express *sfGFP-2ams*, *sfGFP-3ams*, *sfGFP-4ams*, *sfGFP-5ams*, and *sfGFP-6ams* gene products, with the given protein amount obtained from AzFRS-MS which is greater than that obtained from AzFRS (Figure 3B; Supplementary Figure S1). Furthermore, the full-length *sfGFP-7ams*, *sfGFP-8ams*, and *sfGFP-10ams* gene products can be expressed after introducing K431M/A441S double mutations to AzFRS CTD (Figure 3B; Supplementary Figure S1). Although AzFRSc shows slightly better amber read-through efficiency than AzFRS-MS through the observation of *sfGFP-27am* gene product generation, AzFRSc does not generate the full-length *sfGFP-3ams* and *sfGFP-8ams* gene products (Supplementary Figure S2). Therefore, AzFRS-MS is selected for the characterization and its Ftn-**4** protein production is examined. To further confirm the ncAA screening results of AzFRS-MS, sfGFP-**1**∼**9** are respectively expressed and purified and then subjected to ESI-MS analysis. The observed mass of all nine sfGFP proteins generated by AzFRS-MS matches the calculated mass of the full-length sfGFP (Table 2; Figure 3C; Supplementary Figures S3–S11).

**TABLE 2:** ESI-MS analysis of sfGFP and ferritin proteins that produced by AzFRS-MS•tRNA^Pyl^ pair.

Protein^a^	Calculated Mass (Da)	Found Mass (Da)	ESI-MS^b^
sfGFP-**1**	28,096, 27,965 (–Met)^c^	28,096, 27,965	S3
sfGFP-**2**	28,049, 27,918 (–Met)	28,049, 27,918	S4
sfGFP-**3**	28,005, 27,874 (–Met)	28,005, 27,874	S5
sfGFP-**4**	28,011, 27,880 (–Met)	28,011, 27,880	S6
sfGFP-**5**	28,024, 27,893 (–Met)	28,024, 27,893	S7
sfGFP-**6**	28,000, 27,869 (–Met)	28,000, 27,869	S8
sfGFP-**7**	27,984, 27,853 (–Met)	27,984, 27,853	S9
sfGFP-**8**	27,864 (–Met)	27,862	S10
sfGFP-**9**	28,015	27,986^d^	S11
Ftn-81-**4**	22,200, 22,174^e^	22,199, 22,174^e^	S12
Ftn-143-**4**	22,219	22,219	S13
Ftn-2am-**4**	22,261, 22,235^e^	22,261, 22,233^e^	S14
Ftn-81-Cy5	23,207, 22,174^e^	23,207, 23,223^f^, 22,172^e^	S15
A-Ftn-81-**4**	23,448, 23,422^e^	23,451, 23,426^e^	S16
A-Ftn-81-DOX	24,525, 23,422^e^	24,524, 23,421^e^	S17

a All sfGFP proteins are produced from the *sfGFP-27am* gene in GMML medium.

b Full-length sfGFP electrospray and deconvoluted mass spectra.

c (–Met) indicates full-length sfGFP protein without N-terminus methionine residues.

d The found mass of 27,986 Da corresponds to the full-length sfGFP with nitro group reduction, i.e., with an additional amino group, at F27 position and without N-terminal methionine (calculated mass: 27,985 Da).

e These found masses correspond to the full-length Ftn with azide group reduction, which is converted to an amino group.

f An oxygen adducts on Ftn-81-Cy5.

### Ferritin conjugate preparation and chemical moiety loading using SPAAC

**FIGURE 4:**
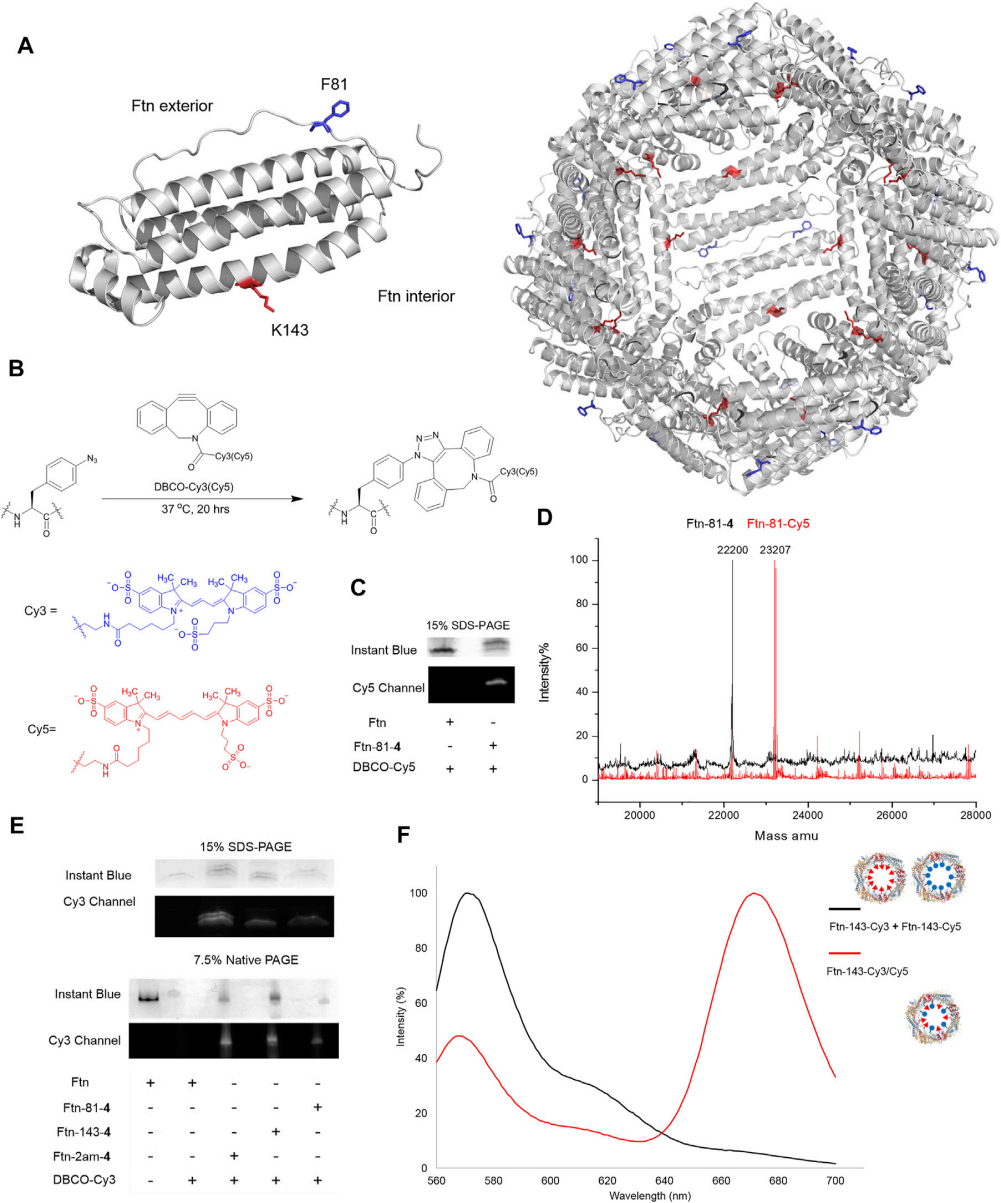
Design of Ftn conjugates and click reaction study for the loading of Cy3 and Cy5. **(A)** Structure of Ftn monomer and the bisection of self-assembled 24mers. F81, facing the exterior of the Ftn nanoparticle, is shown in red stick; K143, facing the interior of the Ftn nanoparticle, is shown in blue stick. The PDB entry is 2FHA. **(B)** Synthesis scheme of SPAAC for loading DBCO-Cy3 (*λ*_ex_/*λ*_em_ = 555/570 nm) and DBCO-Cy5 (*λ*_ex_/*λ*_em_ = 640/664 nm) on Ftn-**4** proteins. **(C)** SPAAC of Ftn-81-**4** and DBCO-Cy5. SDS-PAGE is visualized by Instant Blue staining and Cy5. **(D)** The deconvoluted ESI-MS spectra of Ftn-81-**4** and Ftn-81-Cy5. The calculated and found molecular weights are listed in Table 2. **(E)** The SDS-PAGE and native PAGE analysis of Ftn-Cy3 conjugates. The gels are visualized by Instant Blue staining and Cy3 fluorescence. Twenty micromolars of Ftn protein and 1 mM DBCO-Cy3 are reacted in room temperature for 4 h. **(F)** FRET analysis of Ftn-dye conjugates with the excitation at 550 nm. The black line denotes Ftn-143-Cy3 and Ftn-143-Cy3 protein mixture, whereas the red line denotes Ftn-143-Cy3/Cy5 protein.

The Ftn consists of 24 subunits and can self-assemble into a spherical structure with the outer diameter of 12 nm. The sites for the chemical moiety loading, i.e., a fluorophore or a drug with the molecular weight less than 1,000 Da, are selected based on the x-ray crystal structure of Ftn (PDB code: 2FHA, Figure 4A). The residues F81 pointed outward (labeled in blue in Figure 4A) and K143 pointed inward (labeled in red in Figure 4A) on Ftn are chosen to estimate drug loading efficiency based on the penetration of small molecules into the Ftn cavity and the SPAAC reaction. *Ftn-F81am*, *Ftn-K143am*, and *Ftn-2ams* gene products supplemented with AzFRS-MS and AzF (**4**) produce Ftn-81-**4**, Ftn-143-**4**, and Ftn-2ams-**4**, respectively, and these products are characterized by ESI-MS spectrometry. The measured molecular weight of those products matches their corresponding calculated molecular weight (Table 2; Supplementary Figures S12–14). It is found that, for Ftn-81-**4** and Ftn-2ams-**4**, azide reduction occurs, followed by the conversion to an amino group (Table 2).

DBCO-Cy3 and DBCO-Cy5 (molecular weight: 980.28 and 992.28 Da, respectively) are used to probe the labeling efficiency of the SPAAC reaction in loading DBCO-dye (Figure 4B). The quantitative labeling of Ftn-81-**4** with DBCO-Cy5 is conducted with the 1:5 M ratio. The SDS-PAGE analysis shows that Ftn is not being labeled after DBCO-Cy5 treatment while Ftn-81-**4** gives the bioorthogonality upon DBCO-Cy5 treatment as a clear band can be visualized (Figure 4C). For Ftn-81-**4**, after 4 h treatment with DBCO-Cy5, a species with about 1.0 kDa mass shift is noted from the deconvoluted ESI-MS spectrum, which indicates that Cy5 is labeled through the azido group on Ftn-81-**4**. With the reaction time of 16 h, Ftn-81-**4** (found molecular weight: 22,220 Da; calculated molecular weight: 22,220 Da) is found to be completely converted into Ftn-81-Cy5 (found molecular weight: 23,207 Da; calculated molecular weight: 23,207 Da). A slight azide reduction on Ftn-81-**4** turning into an amino group is observed (22,173 Da); in addition, an oxygen adduct of Ftn-81-Cy5 (23,223 Da) is found (Table 2; Figure 4D; Supplementary Figures S12, S15).

In consideration of structural disruption and disassembling, DBCO-Cy3 labeling on Ftn, Ftn-81-**4**, Ftn-143-**4**, and Ftn-2am-**4** for 4 h is tested; the corresponding labeling efficiency and assembling ratio are then analyzed by SDS-PAGE and native PAGE (Figure 4E). It is noted that Ftn is not being labeled after the treatment with DBCO-Cy3 and remains assembled but shows less protein amount after treating with DBCO-Cy3, which might be resulted from the aggregation. Relatively, Ftn-81-**4**, Ftn-143-**4**, and Ftn-2am-**4** proteins give shifted fluorescent bands on SDS-PAGE and clear fluorescent bands of the assembled protein on native PAGE upon being labeled (Figure 4E). On the SDS-PAGE, Ftn-2am-**4** gives two bands that represent single- and double-labeled Cy3 adducts.

With the successful SPAAC reaction in the DBCO-Cy5 and DBCO-Cy3 labeling on Ftn-81-**4** Ftn-143-**4**, and Ftn-2am-**4**, respectively, the capacity for the incorporation of different and multiple molecules/drugs is subjected to be evaluated. As the inner diameter of Ftn is 8 nm, Cy3 and Cy5 dyes inside Ftn deem to be a suitable fluorescence resonance energy transfer (FRET) pair (Roy et al., 2008) to probe adjacent Cy3/Cy5 within a 70-Å distance. Ftn-143-**4** is selected and treated with DBCO-Cy3 (*λ*_ex_/*λ*_em_ = 555/570 nm) and DBCO-Cy5 (*λ*_ex_/*λ*_em_ = 640/664 nm) dyes at the 1:2.5:2.5 M ratio for 16 h. FRET of the Ftn-143-Cy3 and Ftn-143-Cy5 mixture (1:1; as the control experiment) and Ftn-143-Cy3/Cy5 are measured (Wu and Brand, 1994a; Wu and Brand, 1994b) to ensure that both Cy3 and Cy5 molecules can be co-incorporated into one cage (Figure 4F). After the excitation at 550 nm, the mixture of Ftn-143-Cy3 and Ftn-143-Cy5 (1:1) gives an emission peak at 572 nm but lacks the emission peak at 664 nm, which supports this control system. In contrast, after the excitation at 550 nm on Ftn-143-Cy3/Cy5, a low emission peak at 569 nm is observed but an extra high emission peak at 673 nm arises, which demonstrates the successful FRET and proves that both Cy3 and Cy5 are co-incorporated into the Ftn cavity (Figure 4F).

### Preparation of Ftn targeting Her2^+^ breast cancer cells

**FIGURE 5:**
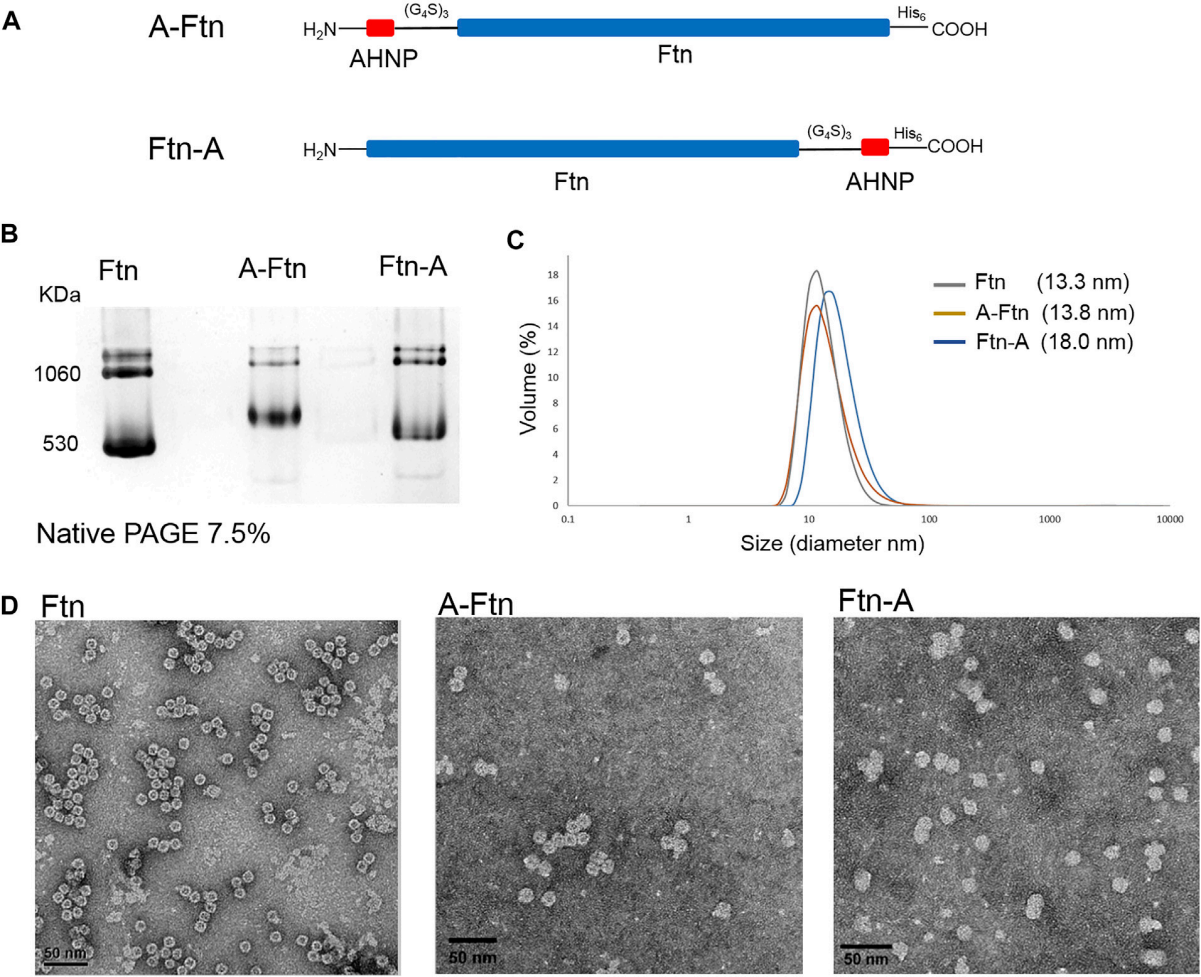
Structural effects from the fused AHNP peptide on Ftn. **(A)** Illustration of A-Ftn and Ftn-A protein constructs. AHNP is the anti-Her2/neu peptide. (G_4_S)_3_ denotes a triple GGGGS peptide sequence. His_6_ indicates C-terminal Histag. **(B)** Assembling analysis of Ftn, A-Ftn, and Ftn-A proteins. Proteins are analyzed by 7.5% native PAGE and visualized by Instant Blue staining. **(C)** DLS analysis of Ftn and AHNP fused Ftn. The protein concentration is set to be 0.5 mg/ml in 50 mM Tris–HCl and 100 mM NaCl at pH 7.4. **(D)** TEM images of Ftn, A-Ftn, and Ftn-A. Scale bar represents 50 nm. The protein concentration is set to be 50 μg/ml in 50 mM Tris–HCl at pH 8.0.

To equip Ftn with a moiety targeting Her2^+^ breast cancer cells, Ftn is fused with AHNP through a non-structural triple GGGGS peptide at one of the two termini (Figure 5A). Since the fused AHNP may affect the self-assembling of Ftn, leading to twisted cavities, the structure analysis of the purified A-Ftn and Ftn-A is conducted using native PAGE, DLS, and TEM, respectively. Native PAGE analysis on A-Ftn and Ftn-A shows that they have similar assembled sizes and patterns, but their sizes are slightly larger than Ftn (Figure 5B). DLS analysis indicates that A-Ftn shares a similar outer diameter with Ftn (13.8 and 13.3 nm, respectively) (Figure 5C); TEM analysis shows that they both share a spherical shape (Figure 5D). However, DLS analysis shows that Ftn-A possesses a larger outer diameter of 18.0 nm (Figure 5C) and the TEM image shows that Ftn-A is irregular in its shape (Figure 5D). Thus, the A-Ftn construct is selected to target the HER2 receptor as it is more structurally suitable.

**FIGURE 6:**
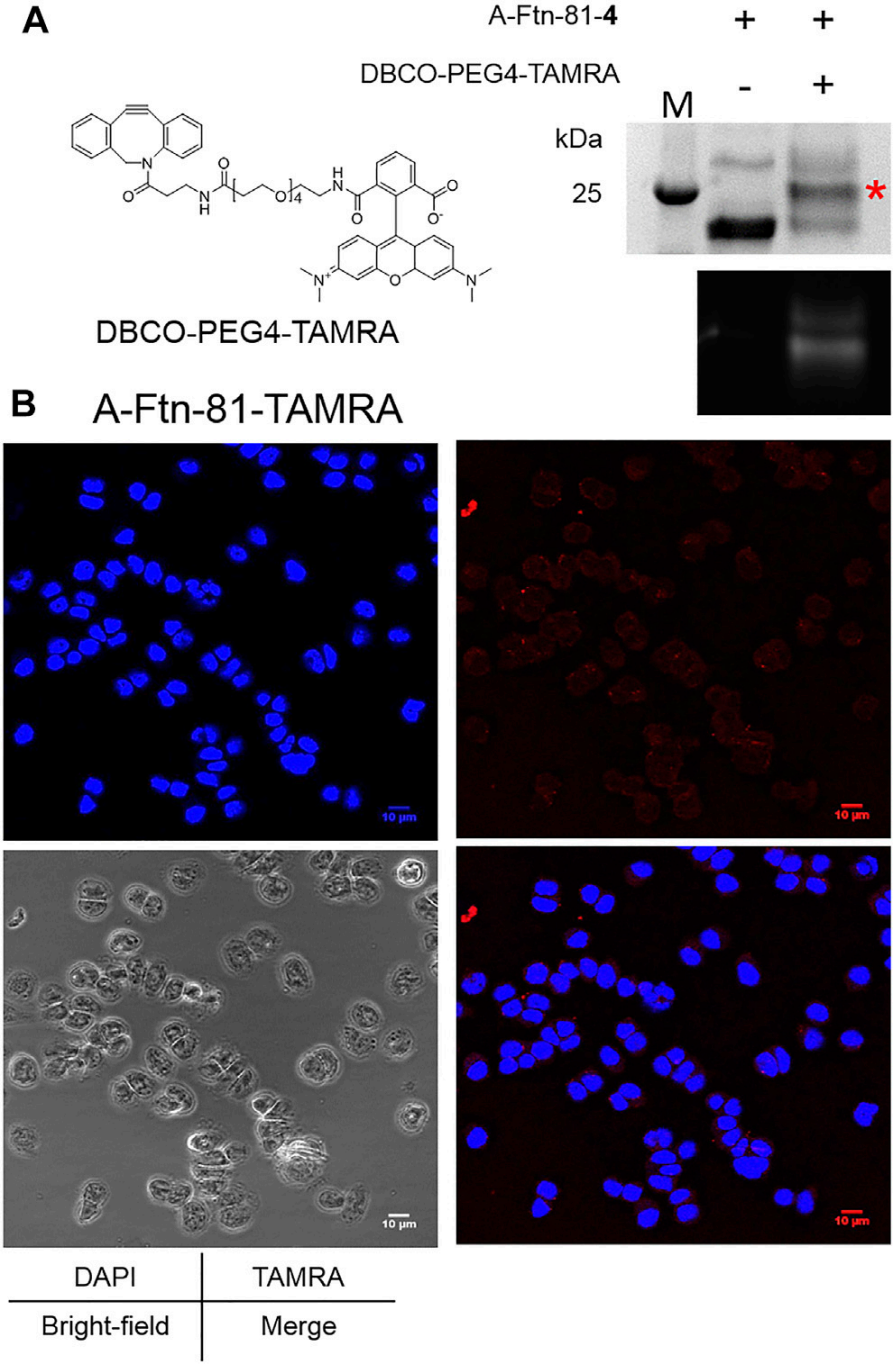
A-Ftn-TAMRA conjugates treatment indicates Her2^+^ BT474 cell targeting ability. **(A)** Chemical structure of DBCO-PEG4-TAMRA and SPAAC labeling analysis after the treatment of A-Ftn-81-4 with DBCO-PEG4-TAMRA. Proteins are analyzed by 15% SDS-PAGE with Instant Blue staining **(upper panel)** and gel fluorescence emission image **(lower panel)**. The red asterisk indicates the TAMRA-labeled Ftn band. M denotes the protein molecular weight marker. **(B)** Confocal microscopy fluorescence image analysis of BT474 cells incubated with 2 μM A-Ftn-81-TAMRA for 1 h. The cell nuclei are stained by DAPI (blue); A-Ftn-81-TAMRA is detected by TAMRA (*λ*_ex_/*λ*_em_ = 553 nm/563 nm, red).

A-Ftn-81-**4** reacts with DBCO-PEG4-TAMRA (Figure 6A) at 1:5 M ratios for 4 h to form A-Ftn-81-TAMRA. SDS-PAGE analysis is performed after the reaction, which gives a fluorescent band with a larger size, supporting that A-Ftn is being labeled (Figure 6B). The effect of A-Ftn on the targeting of the HER2 receptor is evaluated using the BT474 breast cancer cell line. BT474 cells are treated with A-Ftn-81-TAMRA for 1 h and then observed by confocal microscopy. The fluorescence images obtained from confocal microscopy analysis indicate that A-Ftn-81-TAMRA can be found in the cytosol of BT474 cells, suggesting that A-Ftn-81-TAMRA can enter through the cell membrane following the recognition of AHNP by HER2 receptors (Figure 6B).

### Synthesis of A-Ftn–Dox conjugates used in the treatment with BT474 cells

**FIGURE 7:**
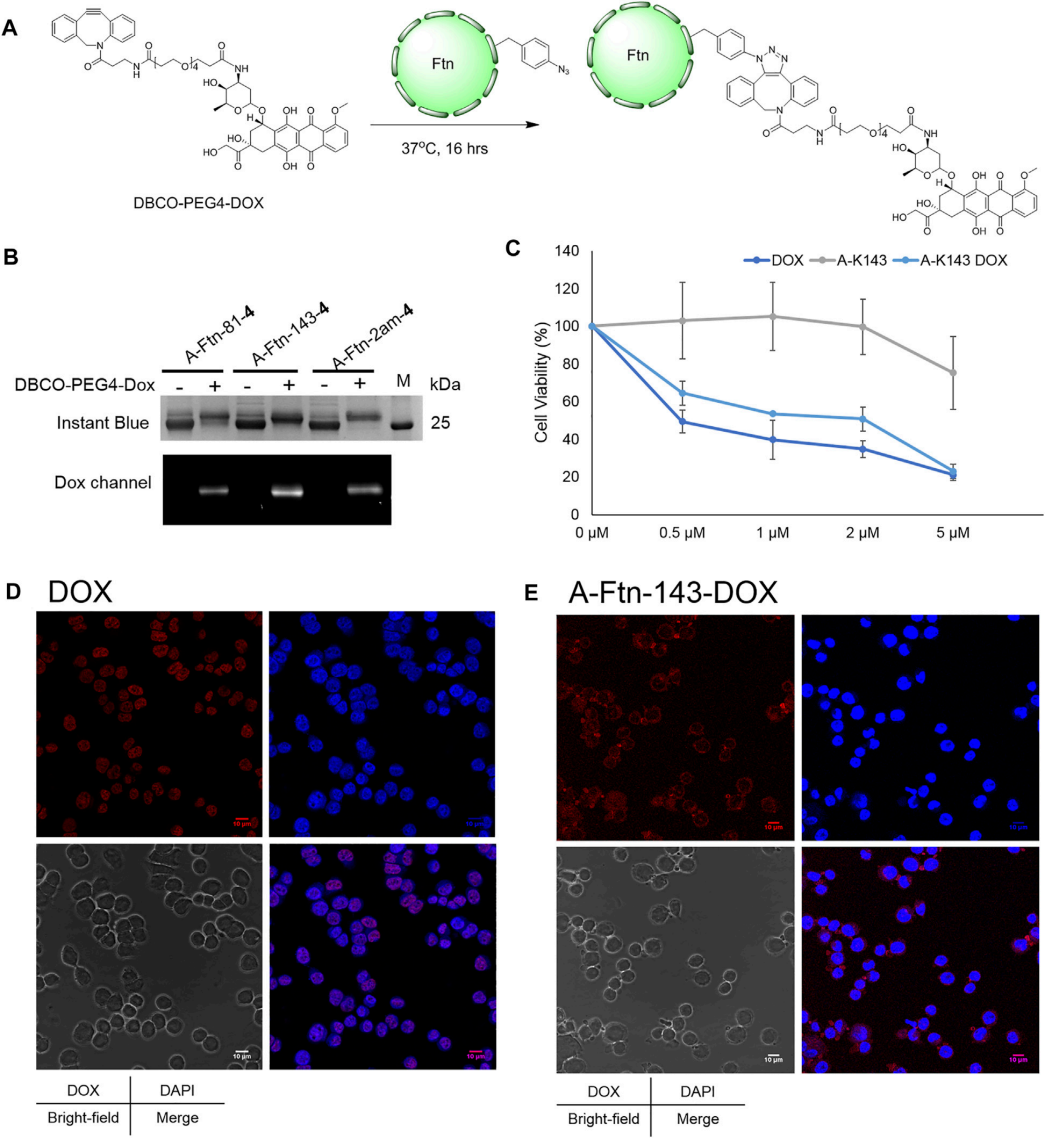
Ftn–Dox conjugates target HER2 overexpressed breast cancer cells. **(A)** Synthesis scheme of Ftn–Dox conjugates. **(B)** SPAAC labeling analysis on A-Ftn-81-**4**, A-Ftn-143-**4**, and A-Ftn-2am-**4** by DBCO-PEG4-DOX. Proteins are analyzed by 15% SDS-PAGE with Instant Blue staining **(upper panel)** and gel fluorescence image **(lower panel)**. M denotes the protein molecular weight marker. **(C)** MTT assay analysis after treating with various concentrations of free Dox, A-Ftn-143-**4** (A-K143), and A-Ftn-143-DOX (A-K143 DOX) for 72 h. Confocal microscopy fluorescence image analysis of BT474 cells incubating with **(D)** 2 μM free Dox and **(E)** 2 μM A-Ftn-143-DOX treating for 1 h. The cell nuclei are visualized by DAPI staining (blue) and by gel fluorescence emission image through DOX (*λ*_ex_/*λ*_em_ = 480 nm/590 nm, red).

Upon the successful demonstration for the capability of A-Ftn entering into BT474 cells, a chemotherapeutic drug, Dox, is loaded into A-Ftn to test the cell targeting and the drug release (Figure 1). The DBCO-PEG4-DOX is synthesized through the NHS-amine coupling between DBCO-PEG4-NHS and Dox. Using the SPAAC reaction, A-Ftn-81-DOX, A-Ftn-143-DOX, and A-Ftn-2am-DOX are synthesized by mixing A-Ftn-81-**4**, A-Ftn-143-**4**, and A-Ftn-2am-**4** with DBCO-PEG4-DOX, respectively, for 16 h (Figure 7A). SDS-PAGE analysis shows that all three A-Ftn proteins are successfully conjugated with DOX and yield an almost complete conversion into A-Ftn–DOX products (Figure 7B). ESI-MS spectrometry is employed to characterize A-Ftn-81-**4** and A-Ftn-81-DOX, respectively. The results show that the protein mass matches the corresponding calculated protein mass, leading to the confirmation of quantitative labeling with the reaction time of 16 h (Table 2, Supplementary Figures S14, S15).

Furthermore, an A-Ftn-143-DOX conjugate is chosen for the evaluation of the capability in entering BT474 cells. The short-term (1 h) and long-term (72 h) treatment of A-Ftn-143-DOX with BT474 cells are examined, with the free Dox as the control treatment. MTT cell viability assay is conducted to examine the cell toxicity for BT474 cells after the treatment with free Dox, A-Ftn-143-**4**, and A-Ftn-143-DOX, respectively, for 72 h. A-Ftn-143-DOX gives a compatible trend as free Dox at the concentration from 0.5 to 5 μM while A-Ftn-143-**4** presents a negligible toxicity effect at this concentration range (Figure 7C). For the short-term (1 h) treatment of free Dox with BT474 cells, it is noted from the merged DAPI and Dox images that free Dox is found in the nucleus (Figure 7D). Nevertheless, for the short-term (1 h) treatment of A-Ftn-143-DOX with BT474 cells, Dox is mainly distributed in the cytosol of BT474 cells (Figure 7E), which suggests the elongated drug release. The slight Dox signal is observed in the nucleus, which resulted from free Dox molecules released from A-Ftn-143-DOX. After the long-term (36 h) treatment, free Dox released from A-Ftn-143-DOX is found to reside in the nucleus (Supplementary Figure S18).

## Discussion

In this study, efficient *Mm*PylRS is developed to charge a chemical moiety that allows the click reaction to occur on the desired delivery system, *i.e.*, the assembled Ftn. This facilitates the future application in the effective fluorophores/drug loading as well as target-specific chemical moiety release (Figure 1). Through the engineering of *Mm*PylRS charging IF (**1**) (Scheme 1), three evolved *Mm*PylRS variants, i.e., IFRS1, IFRS2, and AzFRS, obtained from directed evolution (Wang et al., 2011) are tested against nine *para*-substituted phenylalanine ncAAs (Scheme 1) for substrate range study (Figure 2A). Among these ncAAs, although only **4** that harbors an azide unit is selected for the examination using the SPAAC click reaction herein, some other substrates are also promising in the application in other types of click reaction. For instance, the ncAA **5**, which harbors a propargyl unit, can be employed when using the CuAAC click reaction.

With the conserved mutations at N346 and C348, and combined with other mutation sites, the evolved *Mm*PylRS, IFRS1 and IFRS 2 (Table 1), are found to be specific for certain substrates (Figure 2A). Another evolved *Mm*PylRS variant, AzFRS, with an additional conserved mutation, W417L, that plays as a gate keeper shows the largely enhanced activity for **6** and the mildly enhanced activity for **1**–**5** (Figure 2A). Upon the removal of the bulky indole ring-side chain on W417, the *para*-substituted iodo group can be perfectly harbored in the reshaped binding pocket of the active site (Figure 2B). Through the analysis of the substrate range for wild-type aminoacyl-tRNA synthetases from *E. coli* and evolved PylRS variants, the results show that their binding pockets may generate certain diversity as long as natural amino acids are excluded during the evolution process/history (Ko et al., 2013; Fan et al., 2014; Guo et al., 2014). Since AzFRS shows the enhanced activity toward **4** and **5**, the second sphere engineering of *Mm*PylRS is applied to AzFRS. The rationally designed K431M and D433G mutations, which reside at the CTD, are adapted from the evolved *Mm*PylRS, HarRS (Figures 2C,D) (Mukai et al., 2015). K431M and D433G are suggested to be able to alter the pi–pi stacking cluster among Y242, H432, and F434 by increasing hydrophobicity that may change the interaction with the nearby α-helices and then indirectly improve the activity (Figure 2D). Another A441S mutation at CTD is designed to alter the hydrogen binding network of the R439 guanidino group and to introduce the interaction with the first α-helix of CTD (Figures 2C,E). Through the examination of these single mutations on AzFRS, the results show that AzFRS-S can enhance the activity in charging a variety of substrates while AzFRS-M and AzFRS-G only slightly improve the activity for certain substrates (Figure 3A). Based on the result obtained from AzFRS with single mutations, double and triple mutations on AzFRS are designed to examine whether the synergic effect can be achieved (Figure 3A). Among them, AzFRS-MS and AzFRSc variants are found to be able to further enhance the activity toward miscellaneous substrates. AzFRS-MS can achieve suppression of 10 sequential TAG stop codons when charging **4**, producing the full-length sfGFP (Figure 3B; Supplementary Figures S1, S2). Compared with AzFRS, perhaps, the AzFRS-MS variant not only enhances the activity in a timely manner but also maintains constant acylated-tRNA concentrations during the protein biosynthesis *in vivo*. Meanwhile, due to the altered local hydrophobic interactions and hydrogen binding network which resulted from K431M and A441S mutations, it is suggested that AzFRS-MS can improve the protein solubility and accelerate the reaction rate. Although only the limited activity can be found on SDS-PAGE when charging **7–9** by monitoring the production of the *sfGFP-27am* gene product, ESI-MS spectrometry of sfGFP-**1**∼**9** confirms that the observed molecular mass matches the corresponding calculated mass (Table 2; Figure 3C; Supplementary Figures S3–S11). Overall, AzFRS-MS presents efficient poly-specificity toward all ncAAs employed in this study through the engineering of *Mm*PylRS CTD.

Using the AzFRS-MS variant, the residues F81 pointed outward and K143 pointed inward on Ftn are proved to be suitable for the incorporation of AzF (**4**), either single-incorporated or double-incorporated. The assembled caged structures (Figure 4A) are not disrupted by the incorporation of **4**. Interestingly, through the test of DBCO-Cy3 or DBCO-Cy5 (∼1.0 kDa) labeling (Figure 4B) on Ftn-81-**4**, Ftn-143-**4**, and Ftn-2ams-**4** proteins, DBCO-Cy3 and DBCO-Cy5 are found to easily enter the interior of Ftn cages, not to cause noticeable structural disturbances and disassembling (Figures 4C,E). This is also supported by the labeling results observed from A-Ftn-81-**4**, A-Ftn-143-**4**, and A-Ftn-2ams-**4** proteins after the treatment of DBCO-DOX (∼1.0 kDa), respectively (Figure 7B). These results suggest that the steric hindrance coming from F81 and K143 on the Ftn protein nanostructure for drug labeling through SPAAC chemistry is negligible. The quantitative labeling of Ftn-**4** by DBCO-fluorophores/drugs (Figures 4A, 7A) is conducted through the SPAAC click reaction at the 1:5 M ratio. Ftn-81-Cy5 (Table 1; Figures 4C,D; Supplementary Figures S12, S14) and A-Ftn-81-DOX (Table 1; Figure 7B; Supplementary Figures S16, S17) proteins are characterized by SDS-PAGE, native PAGE, gel fluorescence imaging, and ESI-MS analyses. The Ftn cage might provide a protective space which shields molecules from redox reaction, hydrolysis, or even degradation. The findings herein imply that the labeling at different loading sites might affect the drug stability, and further investigation can be explored. For some drugs with a shorter half-life, the protein cage might prolong or alter the pharmacokinetics. This work shows that Ftn-**4** can be used to load desired drugs up to 48 units per Ftn cage and specifically labeled at F81 or K143 residue (Figure 4E). The loading of two different chemical moieties into a single Ftn cage is successfully demonstrated through the generation of Ftn-143-Cy3/Cy5 which is co-labeled with Cy3 and Cy5 (1:1) inside of the cage. This result suggests that a variety of chemical moieties can be loaded into one cage, which poses many potential applications in the future, for instance, loading multiple therapeutic agents in the cage to allow cancer combination therapy.

An example is given in this study to show the application of this designed delivery system. The targeting AHNP that recognizes the HER2 receptor is fused at one of the two termini of Ftn (Figure 5A). It is shown that A-Ftn maintains the assembled cage structure, whereas Ftn-A is found to be expanded in size with irregular shapes (Figures 5C,D). It is implied that AHNP fused at the C-terminus of Ftn causes the collapse of Ftn cavity and forms fused balls that are observed by TEM images (Figure 5D). It is observed from the treatment of synthesized A-Ftn-81-TAMRA with BT474 breast cancer cells that, AHNP on A-Ftn-81-TAMRA probably targets HER2 receptors on BT474 cells. Followed by the recognition, labeled Ftn enters BT474 cells and mainly resides in the cytosol. Some aggregated forms are observed, which may indicate the co-localization on the cell membrane and lysosome.

Breast cancer is the leading cause of cancer death globally, especially the Her2^+^ subtype, which quickly spreads and potentially develops into metastatic breast cancer. The strategy described here enables the preparation of an Ftn platform conjugated with drugs to allow targeted therapy and chemotherapy for curing malignant breast cancer. DBCO-PEG4-DOX is incorporated on residues of A-Ftn, pointed either inward or outward (Figures 7A,B). After the treatment of A-Ftn-143-DOX with BT474 cells for 72 h, the MTT assay is performed to examine the cytotoxicity (Figure 7C). A-Ftn-143-DOX is shown to exhibit a similar trend as free Dox. However, free Dox is shown to reside at the nucleus immediately after the incubation for 1 h (Figure 7D). In contrast, after the incubation for 1 h, A-Ftn-143-DOX is shown to mainly locate in the cytosol and slightly distribute in the nucleus (Figure 7E). It is hypothesized that, after the 1-h incubation, Dox observed in the nucleus is released from A-Ftn-143-DOX and the release might be resulted from acidic hydrolysis and protease digestion in lysosomes. After the incubation for 36 h, most Dox released from A-Ftn-143-DOX is located in the nucleus (Supplementary Figure S18). As the hydrolysis and the digestion take time, this might pose the chance for the design of extended-release drugs in the future.

## Conclusion

In conclusion, an evolved *Mm*PylRS variant, AzFRS-MS, can improve the amber suppression efficiency when charging AzF (**4**), resulting in suppression of up to 10 sequential TAG stop codons through the monitoring for the production of the *sfGFP-27am* gene product. The improved activity of AzFRS-MS is also established when charging the other eight ncAAs tested in this study. The charging of **4** on Ftn by AzFRS-MS allows the SPAAC chemistry for DBCO-mediated conjugation to occur, leading to the application on the preparation of Ftn-fluorophore/drug conjugates with the quantitative conversion. Using this approach, the N-terminal AHNP-fused Ftn–DOX conjugate is prepared and shown to be able to target HER2 receptors on BT474 breast cancer cells. Conjugated Dox is then slowly released into the nucleus. This showcase study demonstrates the potential use of this approach in therapeutic areas. In addition, the capability to co-label Cy3 and Cy5 in a single cage suggests that this Ftn delivery platform can harbor different chemical moieties for various future applications such as combination therapy. Taken together, an example is given herein to support that the evolved *Mm*PylRS systems enabling the chemical conjugation on proteins present the potential versatile applications, and they are worth further exploration.

## Data Availability

The datasets presented in this study can be found in online repositories. The names of the repository/repositories and accession number(s) can be found in the article/Supplementary Material.
