# Supplementary material for: Ferritin Conjugates With Multiple Clickable Amino Acids Encoded by C-Terminal Engineered Pyrrolysyl-tRNA Synthetase
Source: Front Chem. 2021 Nov 25;9:779976. doi: 10.3389/fchem.2021.779976 (PMC8655692; doi:10.3389/fchem.2021.779976)
Supplement: Supplementary file 1 [file DataSheet1.pdf]

Ferritin conjugates with multiple clickable amino acids encoded by C-terminal engineered pyrrolysyl-tRNA synthetase

Yi-Hui Wang[1†], Mu-Lung Jian[1,2†], Pei-Jung Chen[1,2], Jo-Chu Tsou[1,2], Le P. Truong[1] and Yane-Shih Wang[1,2,*]

[1] Institute of Biological Chemistry, Academia Sinica, Taipei, Taiwan 11529
[2] Institute of Biochemical Sciences, College of Life Science, National Taiwan University, Taipei, Taiwan 10617
* Correspondence: yaneshihwang@gate.sinica.edu.tw; Tel.: +886-2-27855696 ext. 3050
† These authors have contributed equally to this work and share first authorship.

SUPPLEMENTARY MATERIAL

Table of contents

1. DNA and Protein Sequence..S3

2. Primer Lists..S5

3. Supplementary Table and Figures..S7

1. DNA and Protein Sequence

MmPylRS:
atggataaaaaaccactaaacactctgatatctgcaaccgggctctggatgtccaggaccggaacaattcataaaataaaacaccacgaagt
ctctcgaagcaaaatctatattgaaatggcatgcggagaccaccttgttgtaaacaactccaggagcagcaggactgcaagagcgctcaggc
accacaaatacaggaagacctgcaaacgctgcagggtttcggatgaggatctcaataagttcctcacaaaggcaaacgaagaccagacaa
gcgtaaaagtcaaggtcgtttctgcccctaccagaacgaaaaaggcaatgccaaaatccgttgcgagagccccgaaacctcttgagaatac
agaagcggcacaggctcaaccttctggatctaaattttcacctgcgataccggtttccacccaagagtcagtttctgtcccggcatctgtttcaac
atcaatatcaagcatttctacaggagcaactgcatccgcactggtaaaagggaatacgaaccccattacatccatgtctgcccctgttcaggca
agtgcccccgcacttacgaagagccagactgacaggcttgaagtcctgttaaacccaaaagatgagatttccctgaattccggcaagcctttc
agggagcttgagtccgaattgctctctcgcagaaaaaaagacctgcagcagatctacgcggaagaaagggagaattatctggggaaactcg
agcgtgaaattaccaggttctttgtggacaggggttttctggaaataaaatccccgatcctgatccctcttgagtatatcgaaaggatgggcattg
ataatgataccgaactttcaaaacagatcttcagggttgacaagaacttctgcctgagacccatgcttgctccaaacctttacaactacctgcgc
aagcttgacagggccctgcctgatccaataaaaatttttgaaataggcccatgctacagaaaagagtccgacggcaaagaacacctcgaag
agtttaccatgctgaacttctgccagatgggatcgggatgcacacgggaaaatcttgaaagcataattacggacttcctgaaccacctgggaat
tgatttcaagatcgtaggcgattcctgcatggtctatggggataccctgatgtaatgcacggagacctggaactttcctctgcagtagtcggacc
cataccgcttgaccgggaatggggtattgataaaccctggataggggcaggtttcgggctcgaacgccttctaaaggttaaacacgactttaaa
aatatcaagagagctgcaaggtccgagtcttactataacgggatttctaccaacctgtaa

tRNA$^{Pyl}_{CUA}$:
*ggaaacctgatcatgtagatcgaatggact**cta**aatccgttcagccgggttagattcccggggtttccgcca*
The anticodon CTA is labeled in bold letters

sfGFP:
atgagcaagggcgaagaactgtttacgggcgtggtgccgattctggtggaactggatggtgatgtcaatggtcacaaattcagcgtgcgcggc
gaaggtgaaggcgatgcaaccaatggtaaactgacgctgaagtttatttgcaccacgggtaaactgccggttccgtggccgaccctggtcacc
acgctgacgtatggtgttcagtgtttcagtcgttacccggatcacatgaaacgccacgactttttcaagtccgcgatgccggaaggttatgtccaa
gaacgtaccatctcatttaaagatgacggcacctacaaaacgcgcgccgaagtgaaattcgaaggtgatacgctggttaaccgtattgaactg
aaaggcatcgattttaaggaagacggtaatattctgggccataaactggaatataacttcaattcgcacaacgtgtacatcaccgcagataag
cagaagaacggtatcaaggctaacttcaagatccgccataatgtggaagatggcagcgttcaactggccgaccactatcagcaaaacaccc
cgattggtgatggcccggtcctgctgccggacaatcattacctgagcacgcagtctgtgctgagtaaagatccgaacgaaaagcgtgaccac
atggtcctgctggaattcgtgaccgcggccggcatcacgcacggtatggacgaactgtataaaggctcagagctccatcaccatcaccatcac
taa

Human heavy chain ferritin (Ftn):
atgaccaccgcctctacctcacaggtgcgtcagaattatcatcaggatagtgaagcagcaattaatcgccagattaatctggaactgtatgcaa
gctatgtgtatctgtctatgagctattattttgatcgcgatgatgttgccctgaaaaattttgccaaatattttctgcatcagtctcatgaagaacgcga
acatgccgaaaaactgatgaaattacagaatcagcgtggtggtcgtatttttcttcaagatattaaaaaaccggattgtgatgattgggaaagcg
gcctgaatgcgatggaatgtgccttacatcttgagaaaaatgttaatcagtcactgctggaactgcataaactggcaaccgataaaaatgatcc
gcatctgtgtgattttattgaaacccattatctgaatgagcaggttaaagccattaaagaactgggcgatcatgttaccaatctccgcaaaatggg
cgccccggaaagtggcttagccgaatatctgtttgataaacataccttaggcgatagcgataacgaaagttaa

*Mm*PylRS:
MDKKPLNTLISATGLWMSRTGTIHKIKHHEVSRSKIYIEMACGDHLVVNNSRSSRTARALRHH
KYRKTCKRCRVSDEDLNKFLTKANEDQTSVKVKVVSAPTRTKKAMPKSVARAPKPLENTEA
AQAQPSGSKFSPAIPVSTQESVSVPASVSTSISSISTGATASALVKGNTNPITSMSAPVQASAPAL
TKSQTDRLEVLLNPKDEISLNSGKPFRELESELLSRRKKDLQQIYAEERENYLGKLEREITRFF
VDRGFLEIKSPILIPLEYIERMGIDNDTELSKQIFRVDKNFCLRPMLAPNLYNYLRKLDRALPDP
IKIFEIGPCYRKESDGKEHLEEFTMLNFCQMGSGCTRENLESIITDFLNHLGIDFKIVGDSCMV
YGDTLDVMHGDLELSSAVVGPIPLDREWGIDKPWIGAGFGLERLLKVKHDFKNIKRAARSES
YYNGISTNL
Mutated residues, K431, D433, and A441, are labeled in red color.

sfGFP:
MS^{2}KGEELF^{8}TGVVPILVELDGDVNGHKF^{27}SVRGEGEGDATNGKLTLKF^{46}ICTTGKLPVPWPTL VTTLTYGVQCF^{71}SRYPDHMKRHDFFKSAMPEGYVQERTISF^{100}KDDGTYKTRAEVKF^{114}EGDT LVNRIELKGIDF^{130}KEDGNILGHKLEYNF^{145}NSHNVYITADKQKNGIKANF^{165}KIRHNVEDGSVQ LADHYQQNTPIGDGPVLLPDNHYLSTQSVLSKDPNEKRDHMVLLEF^{223}VTAAGITHGMDELY KGSELHHHHHH
Residues labeled in red represents the positions for ncAA incorporation with amber mutations.

Ftn:
MTTASTSQVRQNYHQDSEAAINRQINLELYASYVYLSMSYYFDRDDVALKNFAKYFLHQSHE EREHAEKLMKLQNQRGGRIF^{81}LQDIKKPDCDDWESGLNAMECALHLEKNVNQSLLELHKLA TDKNDPHLCDFIETHYLNEQVK^{143}AIKELGDHVTNLRKMGAPESGLAEYLFDKHTLGDSDNE S
Residues labeled in red represents the positions for ncAA incorporation with amber mutations.

Anti-HER2/neu peptide (AHNP):
FCDGFYACYMDV

2. Primer lists

Primers	Primer sequence (5′ to 3′)
pET-pylT-sfGFP	
sfGFP-NdeI-F	gagatatacatatgagcaagggcgaag
sfGFP-SacI-R	gatggtgatggagctctgagcctttatac
F27am-R	caccttcgccgcgcacgctctatttgtgacc
F27am-F	ggtcacaaatagagcgtgcgcggcgaaggtg
NdeI-S2am-F	ggagatatacatatgtagaagggcgaagaactg
NdeI-F8am-F	gagatatacatatgagcaagggcgaagaactgtagacggg
F46am-F	caatggtaaactgacgctgaagtagatttgcaccacggg
F46am-R	cccgtggtgcaaatctacttcagcgtcagtttaccattg
F71am-F	cgtatggtgttcagtgttagagtcgttacccggatcac
F71am-R	gtgatccgggtaacgactctaacactgaacaccatacg
F100am-F	caagaacgtaccatctcatagaaagatgacggcacctac
F100am-R	gtaggtgccgtcatctttctatgagatggtacgttcttg
F114am-F	gccgaagtgaaataggaaggtgatacgctggttaaccg
F114am-R	cggttaaccagcgtatcaccttcctatttcacttcggc
F165am-F	gaacggtatcaaggctaactagaagatccgccataatgtg
F165am-R	cacattatggcggatcttctagttagccttgataccgttc
F223am-F	gaaaagcgtgaccacatggtcctgctggaataggtgac
F223am-R	gtcacctattccagcaggaccatgtggtcacgcttttc
pET-pylT-Ftn	
Ftn-NdeI-F	gatatacatatgaccaccgcctctacctcacag
Ftn-SacI-R	gtgatggagctcactttcgttatcgctatcg
F81am-F	ggtggtcgtatttagcttcaagatattaaaaaaccg
F81am-R	cggttttttaatatcttgaagctaaatacgaccacc
K143am-F	gaatgagcaggtttaggccattaaagaactg
K143am-R	cagttctttaatggcctaaacctgctcattc
N-AHNP-F	gagatatacatatgttttgcgatggcttctatgcgtgc
N-AHNP-R	ccaccaccaccactcacatccatatagcacgcatagaagcc
3XG4S-Ftn-F	agtggtggtggtggtagcggagggggaggaagcggaggaggaggcagc
3XG4S-Ftn-R	ctgtgaggtagaggcggtggtgctgcctcctcctcc

C-ANHP-F	gcttttgcgatggcttctatgcgtgctatatggatgtgg
C-ANHP-R	ggtgatggtgatggagctccacatccatatagc
Ftn-3XG4S-C-ANHP-F	gcgataacgaaagtagtggtggtggtggtagcggagggggagg
Ftn-3XG4S-C-ANHP-R	gccatcgcaaaagctgcctcctcctccgcttcctccccctccgc
pCDF-PylRS	
EcoRI-F	gatttccctgaattccggcaagcctttcagggagc
BamHI-R	ggtcgacggatccttacaggttggtagaaatcccgttatag
IFRS1-MLS-F	cttgctccaaacatgttgaactactctcgcaagcttg
IFRS1-MLS-R	caagcttgcgagagtagttcaacatgtttggagcaag
IFRS1-SM-F	gtttaccatgctgtcgttcatgcagatgggatc
IFRS1-SM-R	gatcccatctgcatgaacgacagcatggtaaac
IFRS2-TA-F	gtttaccatgctgtcgttcatgcagatgggatc
IFRS2-TA-R	gatcccatctgcatgaacgacagcatggtaaac
AzFRS-AM-F	gtttaccatgctggcgttcatgcagatgggatc
AzFRS-AM-R	gatcccatctgcatgaacgccagcatggtaaac
AzFRS-L-F	gtattgataaacccсttataggggcaggtttcg
AzFRS-L-R	cgaaacctgcccctataaggggtttatcaatac
K431M-F	ctaaaggttatgcacgactttaaaaatatcaagag
K431M-R	ctcttgatatttttaaagtcgtgcataacctttag
D433G-F	ctaaaggttaaacacggctttaaaaatatcaagag
D433G-R	ctcttgatatttttaaagccgtgtttaacctttag
K431M/D433G-F	ctaaaggttatgcacggctttaaaaatatcaagag
K431M/D433G-R	ctcttgatatttttaaagccgtgcataacctttag
A441S-F	ctttaaaaatatcaagagagcttcaaggtccg
A441S-R	cggaccttgaagctctcttgatatttttaaag
K431M/A441S-F	gttatgcacgactttaaaaatatcaagagagcttcaagg
K431M/A441S-R	ccttgaagctctcttgatatttttaaagtcgtgcataac
D433G/A441S-F	gttaaacacggctttaaaaatatcaagagagcttcaagg
D433G/A441S-R	ccttgaagctctcttgatatttttaaagccgtgtttaac
K431M/D433G/A441S-F	gttatgcacggctttaaaaatatcaagagagcttcaagg
K431M/D433G/A441S-R	ccttgaagctctcttgatatttttaaagccgtgcataac

3. Supplementary Table and Figures

Table S1 sfGFP multiple amber mutation variants employed in this study.

sfGFP	S2	F8	F27	F46	F71	F84	F100	F114	F130	F145	F165	F223
sfGFP-2ams			●	●								
sfGFP-3ams		●			●		●					
sfGFP-4ams			●			●	●	●				
sfGFP-5ams			●			●	●	●			●	
sfGFP-6ams			●			●	●	●			●	●
sfGFP-7ams		●	●			●	●	●			●	●
sfGFP-8ams	●	●	●			●	●	●			●	●
sfGFP-10ams		●	●	●	●		●	●	●	●	●	●

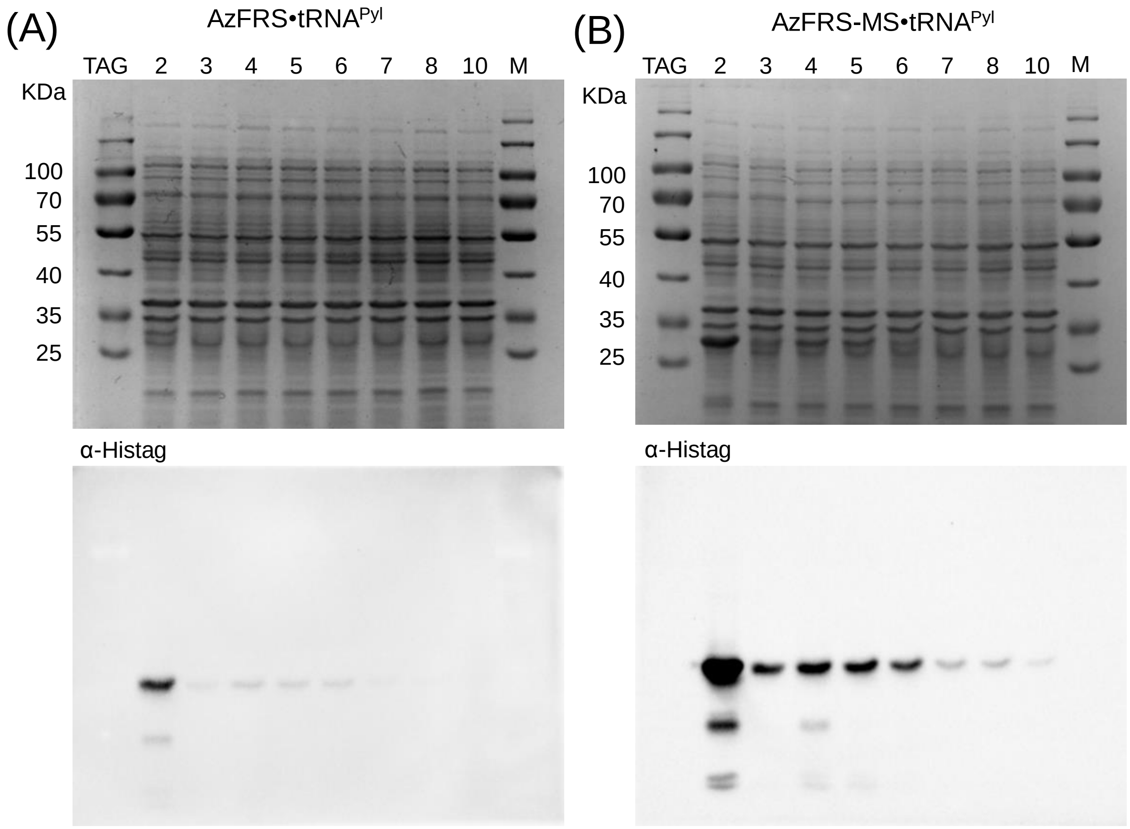

Figure S1. Tandem amber codons suppression efficiency analysis for sfGFP production by AzFRS•tRNA^{Pyl} and AzFRS-MS•tRNA^{Pyl} pairs.
Amber suppression of *sfGFP-2ams~8ams* and *sfGFP-10ams* genes, containing 2~10 TAG stop codons suppression, produce full-length sfGFP proteins with the incorporation of multiple **4**. The sfGFP proteins are overexpressed in *E. coli* BL21 (DE3) coding (A) AzFRS•tRNA^{Pyl} or (B) AzFRS•tRNA^{Pyl} pairs supplemented with 1 mM IPTG and 1 mM **4** in GMML medium at 37°C for 12 hours. The whole-cell lysate is analyzed by SDS-PAGE (upper gel) and Western blotting (lower gel) against anti-Histag antibody (indicating as α-His6X).

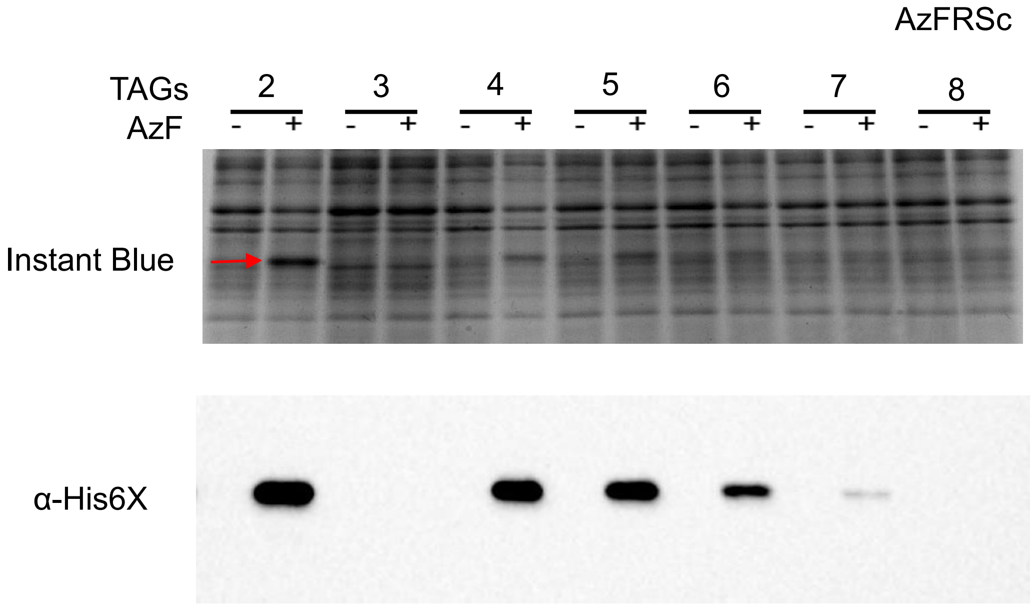

Figure S2. Tandem amber codons suppression efficiency analysis for sfGFP production by AzFRSc•tRNA^{Pyl} pair.
Amber suppression of *sfGFP-2ams~8ams* genes, containing 2~8 TAG stop codons suppression, produce full-length sfGFP proteins with the incorporation of multiple **4**. The sfGFP proteins are overexpressed in *E. coli* BL21 (DE3) coding AzFRSc•tRNA^{Pyl} pair supplemented with 1 mM IPTG and 1 mM **4** in GMML medium at 37°C for 12 hours. The whole-cell lysate is analyzed by SDS-PAGE (upper gel) and Western blotting (lower gel) against anti-Histag antibody (indicating as α-His6X). The red arrow indicates the protein band for sfGFP.

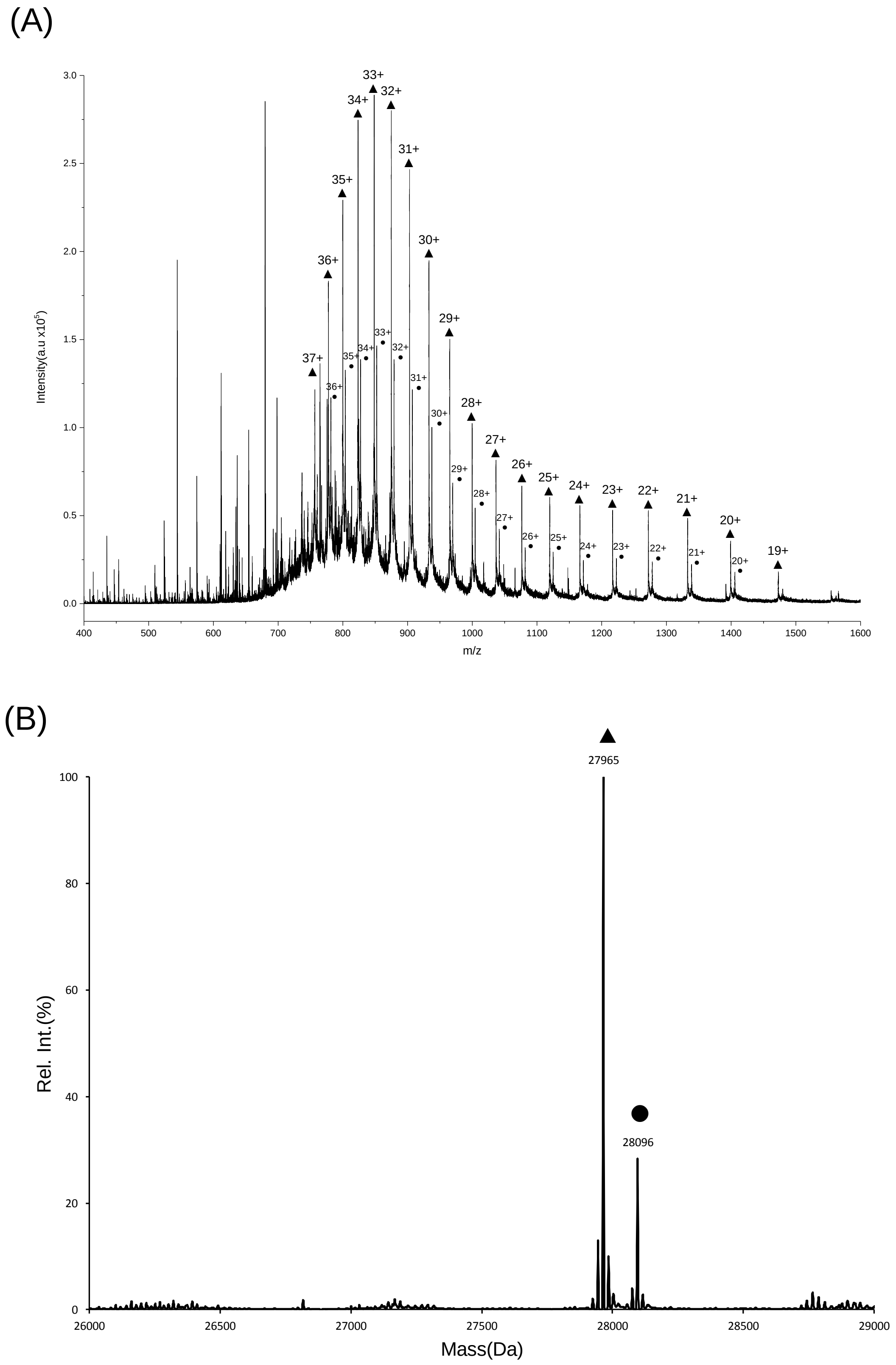

Figure S3. Molecular mass determination of sfGFP-1.
(A) The ESI-MS and (B) the deconvoluted spectra of sfGFP-**1**. Full-length sfGFP-**1** is produced using AzFRS-MS•$tRNA^{Pyl}$ pair in *E. coli* BL21 (DE3) supplemented with 1 mM IPTG and 1 mM **1** in GMML medium. The calculated molecular masses are 28,096 Da and 27,965 Da (-Met); observed molecular masses are 28,096 Da and 27,965 Da (-Met).

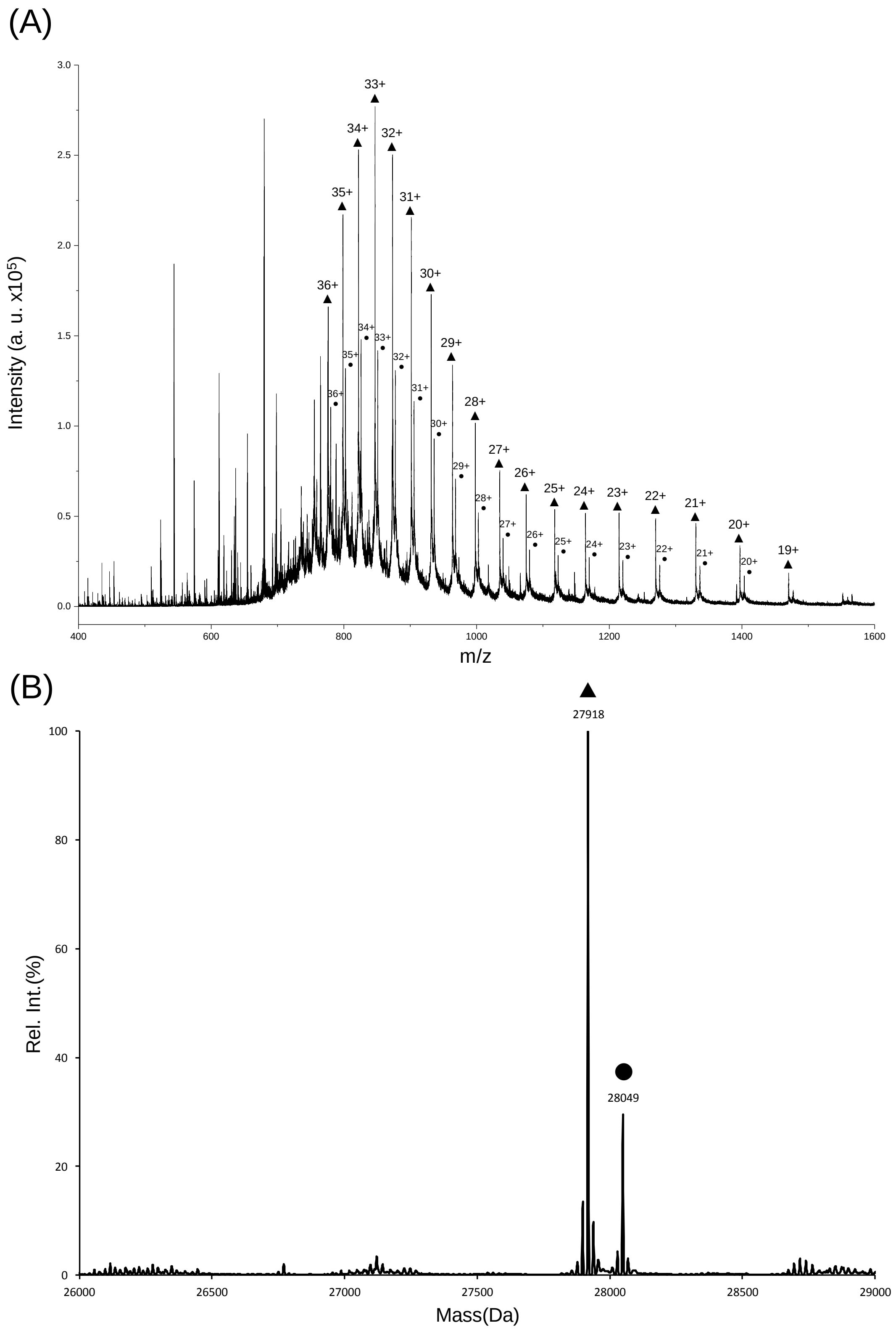

Figure S4. Molecular mass determination of sfGFP-2.
(A) The ESI-MS and (B) the deconvoluted spectra of sfGFP-**2**. Full-length sfGFP-**2** is produced using AzFRS-MS•tRNA^{Pyl} pair in *E. coli* BL21 (DE3) supplemented with 1 mM IPTG and 1 mM **2** in GMML medium. The calculated molecular masses are 28,049 Da and 27,918 Da (-Met); observed molecular masses are 28,049 Da and 27,918 Da (-Met).

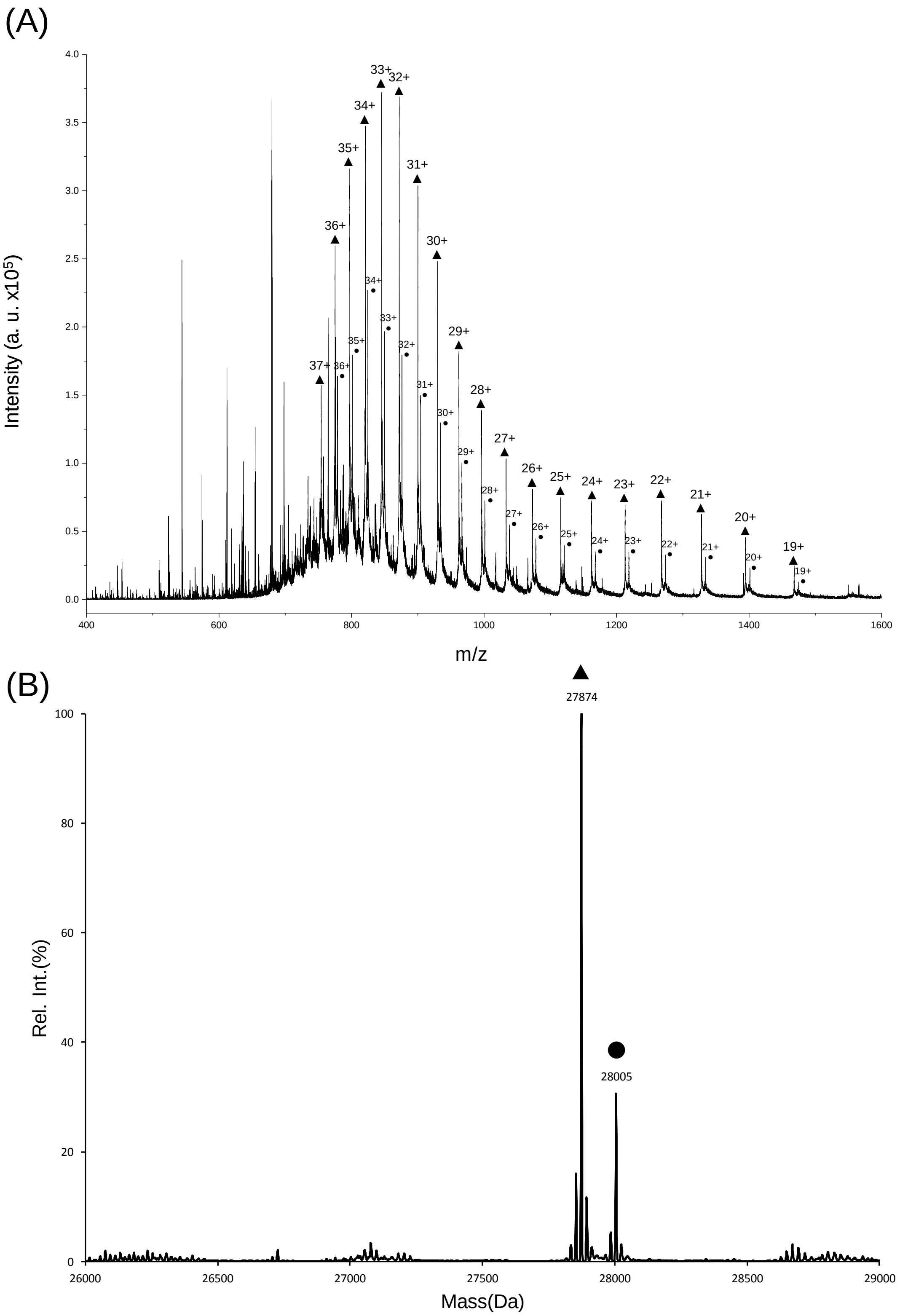

Figure S5. Molecular mass determination of sfGFP-3.
(A) The ESI-MS and (B) the deconvoluted spectra of sfGFP-**3**. Full-length sfGFP-**3** is produced using AzFRS-MS•tRNA^{Pyl} pair in *E. coli* BL21 (DE3) supplemented with 1 mM IPTG and 1 mM **3** in GMML medium. The calculated molecular masses are 28,005 Da and 27,874 Da (-Met); observed molecular masses are 28,005 Da and 27,874 Da (-Met).

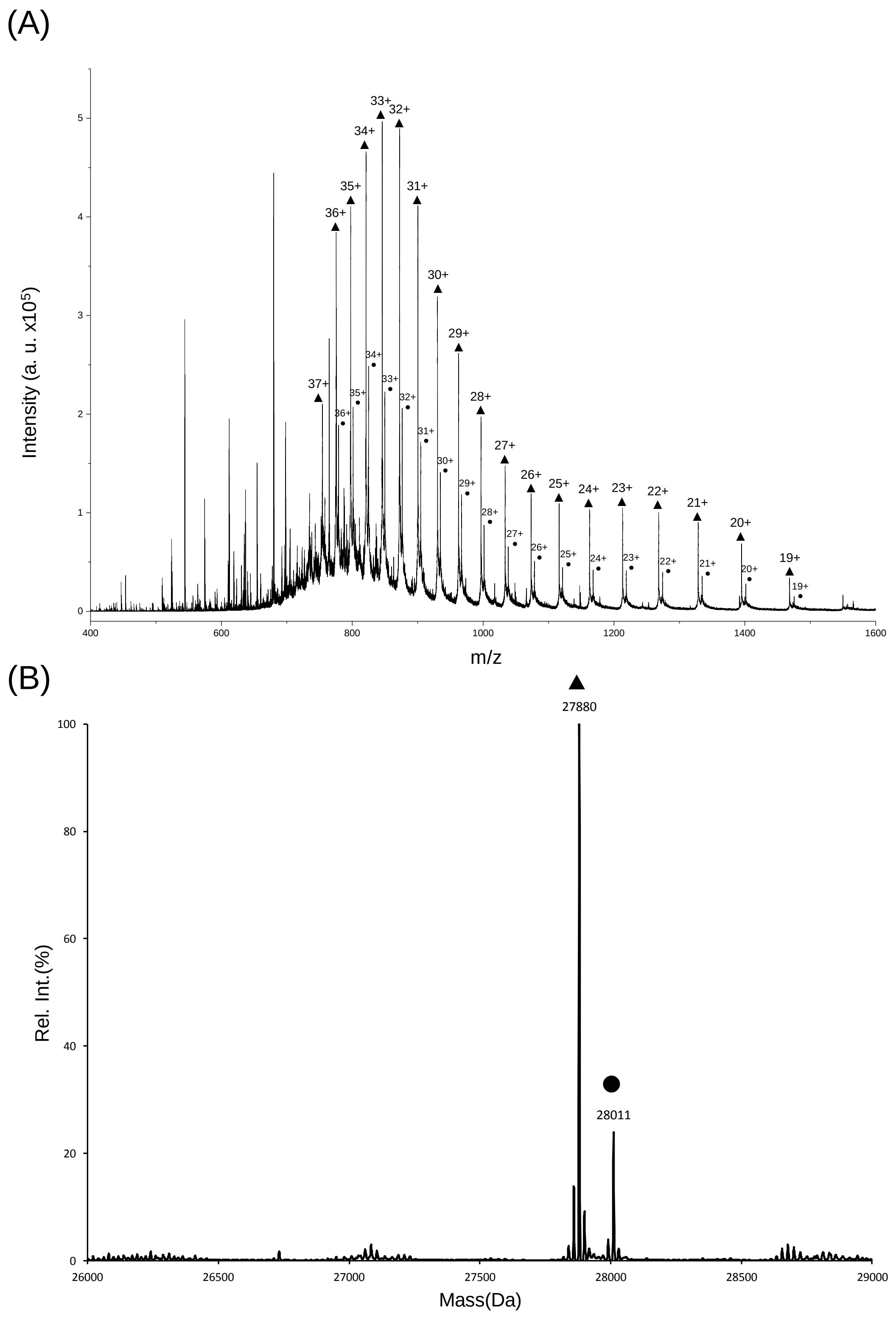

Figure S6. Molecular mass determination of sfGFP-4.
(A) The ESI-MS and (B) the deconvoluted spectra of sfGFP-**4**. Full-length sfGFP-**4** is produced using AzFRS-MS•tRNA^{Pyl} pair in *E. coli* BL21 (DE3) supplemented with 1 mM IPTG and 1 mM **4** in GMML medium. The calculated molecular masses are 28,011 Da and 27,880 Da (-Met); observed molecular masses are 28,011 Da and 27,880 Da (-Met).

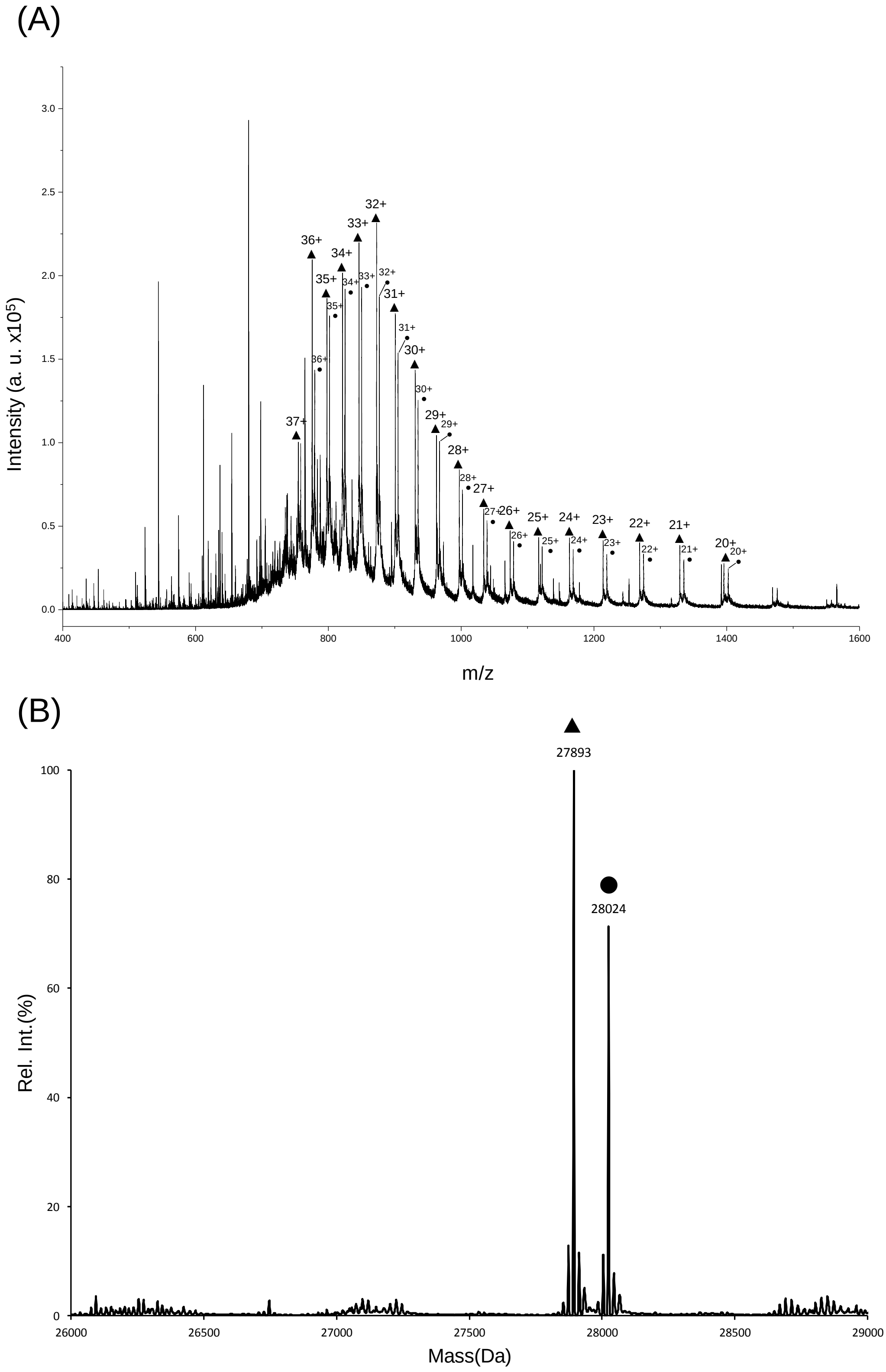

Figure S7. Molecular mass determination of sfGFP-5.

(A) The ESI-MS and (B) the deconvoluted spectra of sfGFP-**5**. Full-length sfGFP-**5** is produced using AzFRS-MS•tRNA^{Pyl} pair in *E. coli* BL21 (DE3) supplemented with 1 mM IPTG and 1 mM **5** in GMML medium. The calculated molecular masses are 28,024 Da and 27,893 Da (-Met); observed molecular masses are 28,024 Da and 27,893 Da (-Met).

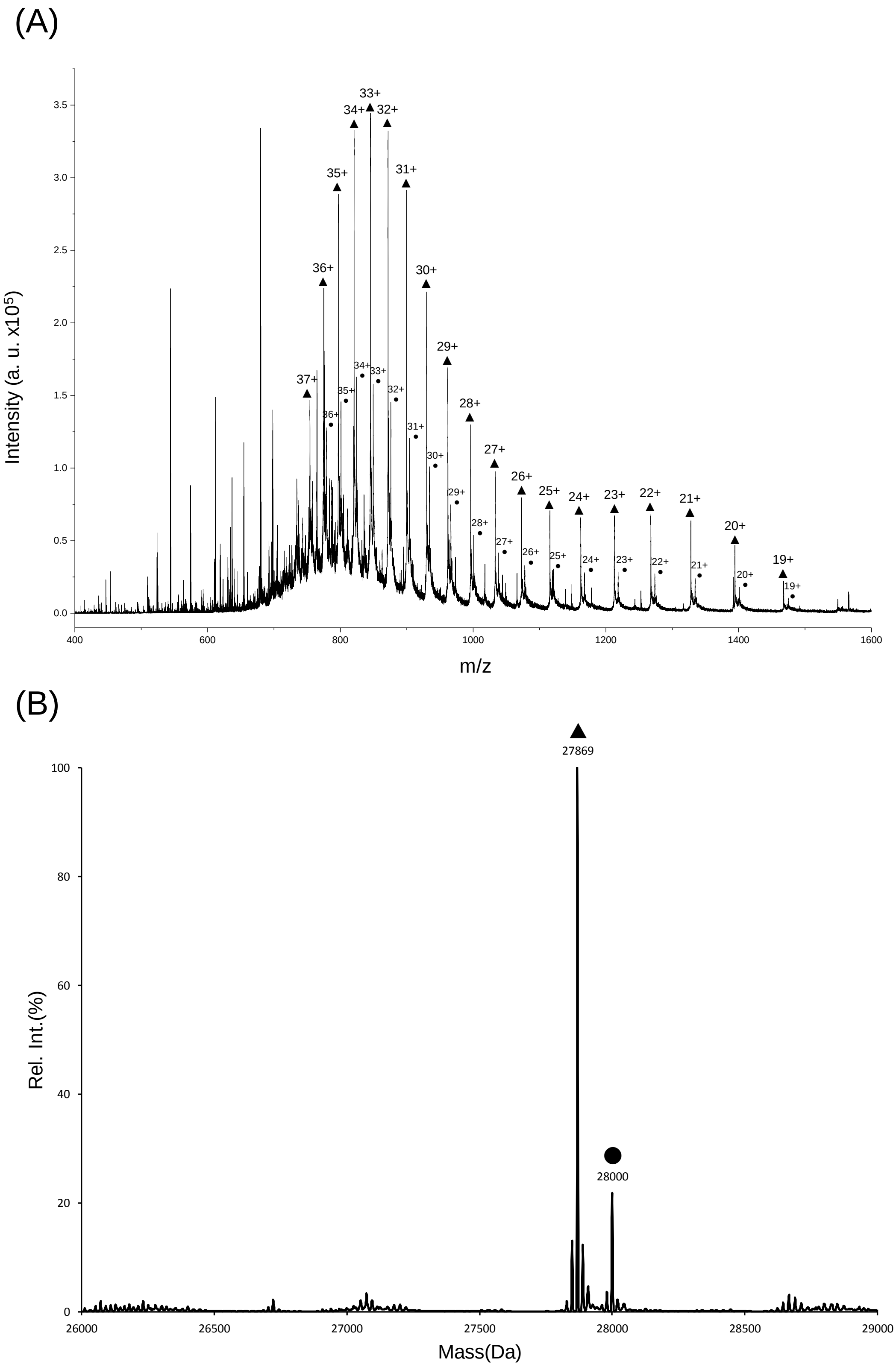

Figure S8. Molecular mass determination of sfGFP-6.

(A) The ESI-MS and (B) the deconvoluted spectra of sfGFP-**6**. Full-length sfGFP-**6** is produced using AzFRS-MS•tRNA^{Pyl} pair in *E. coli* BL21 (DE3) supplemented with 1 mM IPTG and 1 mM **6** in GMML medium. The calculated molecular masses are 28,000 Da and 27,869 Da (-Met); observed molecular masses are 28,000 Da and 27,869 Da (-Met).

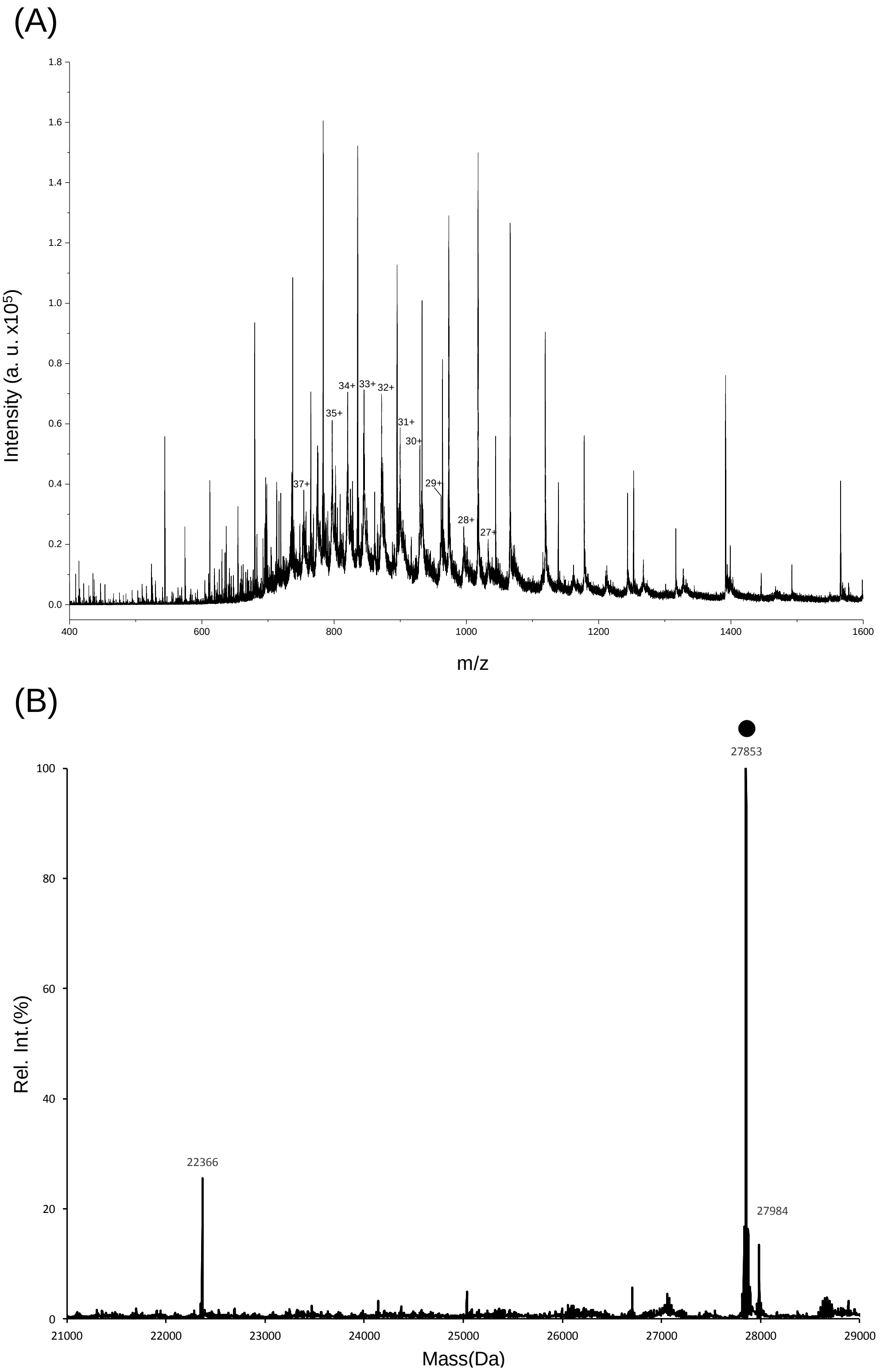

Figure S9. Molecular mass determination of sfGFP-7.

(A) The ESI-MS and (B) the deconvoluted spectra of sfGFP-**7**. Full-length sfGFP-**7** is produced using AzFRS-MS•tRNA^{Pyl} pair in *E. coli* BL21 (DE3) supplemented with 1 mM IPTG and 1 mM **7** in GMML medium. The calculated molecular masses are 27,984 Da and 27,853 Da (-Met); observed molecular masses are 27,984 Da, 27,853 Da (-Met) and 22,366 Da (N-terminal truncated fragment). The charge distribution of peak 27,853 Da (filled circle) is assigned in ESI-MS spectrum.

(A)

(B)

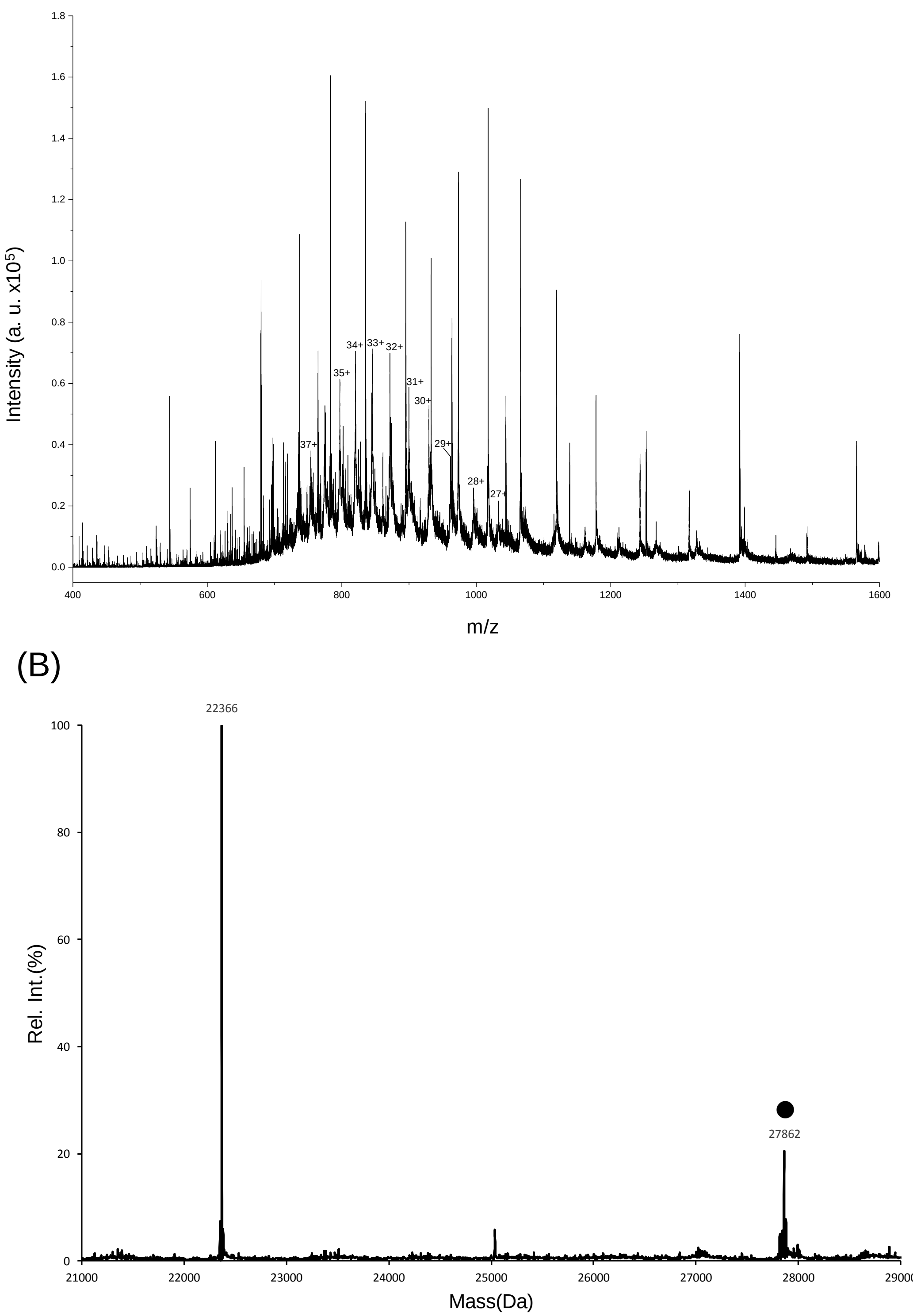

Figure S10. Molecular mass determination of sfGFP-8.
(A) The ESI-MS and (B) the deconvoluted spectra of sfGFP-**8**. Full-length sfGFP-**8** is produced using AzFRS-MS•tRNA^{Pyl} pair in *E. coli* BL21 (DE3) supplemented with 1 mM IPTG and 1 mM **8** in GMML medium. The calculated molecular masse is 27,864 Da (-Met); observed molecular masses are 27,862 Da (-Met) and 22,366 Da (N-terminal truncated fragment). The charge distribution of peak 27,862 Da (filled circle) is assigned in ESI-MS spectrum.

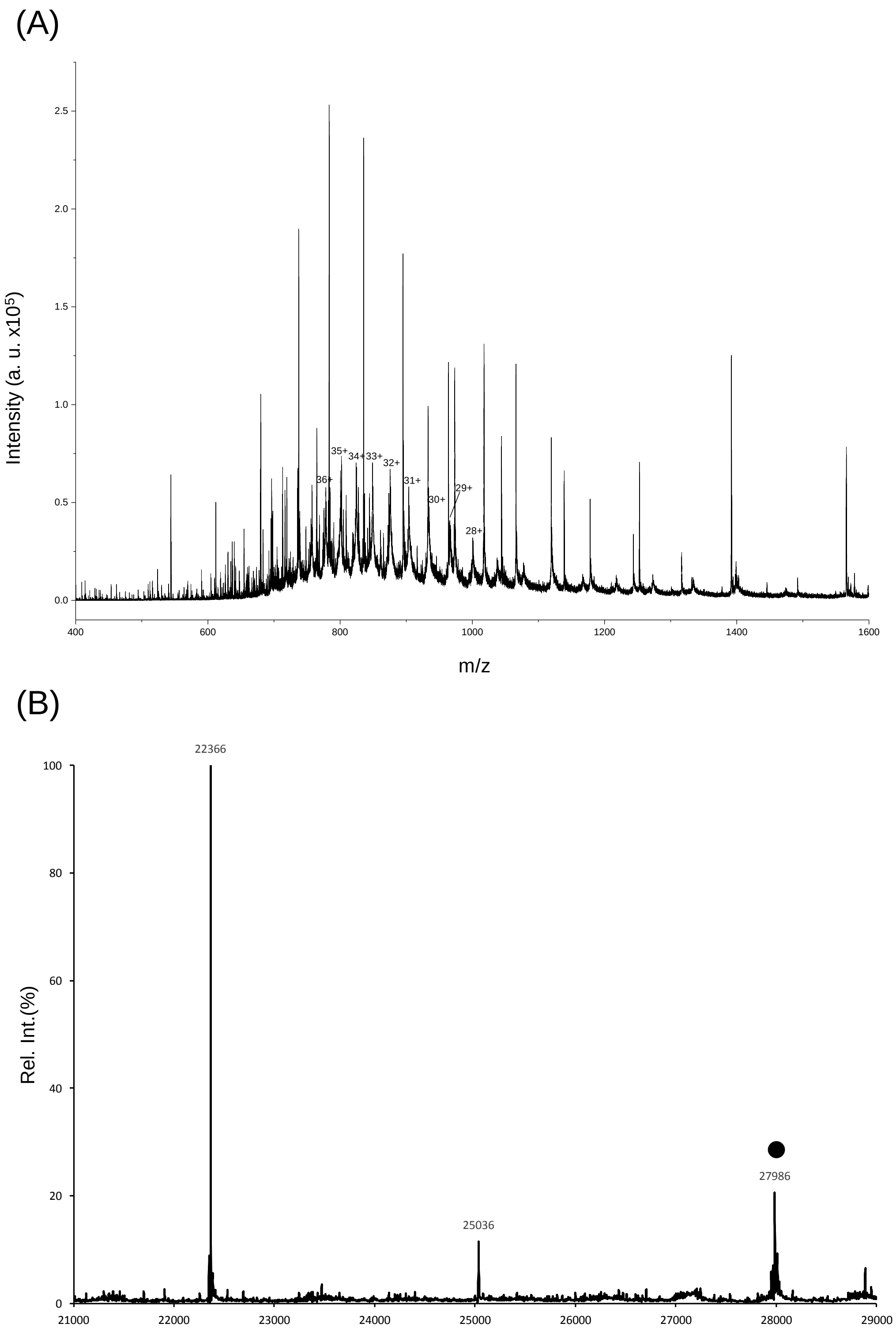

Figure S11. Molecular mass determination of sfGFP-9.
(A) The ESI-MS and (B) the deconvoluted spectra of sfGFP-**9**. Full-length sfGFP-**9** is produced using AzFRS-MS•tRNA^{Pyl} pair in *E. coli* BL21 (DE3) supplemented with 1 mM IPTG and 1 mM **9** in GMML medium. The calculated molecular masse is 28,015 Da; observed molecular masses are 27,986 Da (The nitro group are reduced to amino group.), 25,036 Da and 22,366 Da (N-terminal truncated fragment). The charge distribution of peak 27,986 Da (filled circle) is assigned in ESI-MS spectrum.

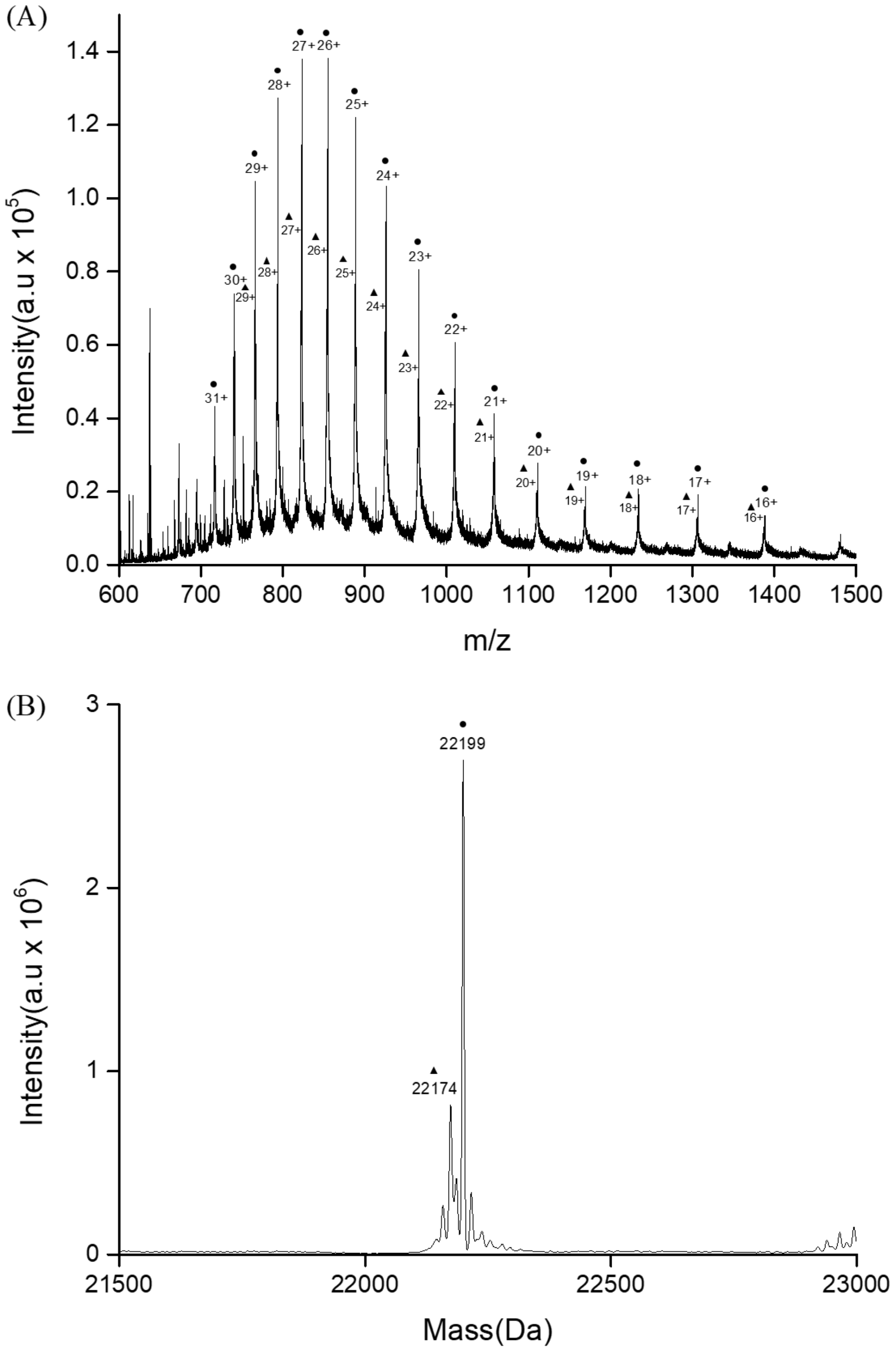

Figure S12. Molecular mass determination of Ftn-81-4.
(A) The ESI-MS and (B) the deconvoluted spectra of Ftn-81-**4**. Full-length Ftn-81-**4** is produced using AzFRS-MS•tRNA^{Pyl} pair in *E. coli* BL21 (DE3) supplemented with 1 mM IPTG and 1 mM **4** in GMML medium. The calculated molecular masse is 22,200 Da; observed molecular masses are 22,199 Da and 22,174 Da (-N_3 converted to -NH_2 group).

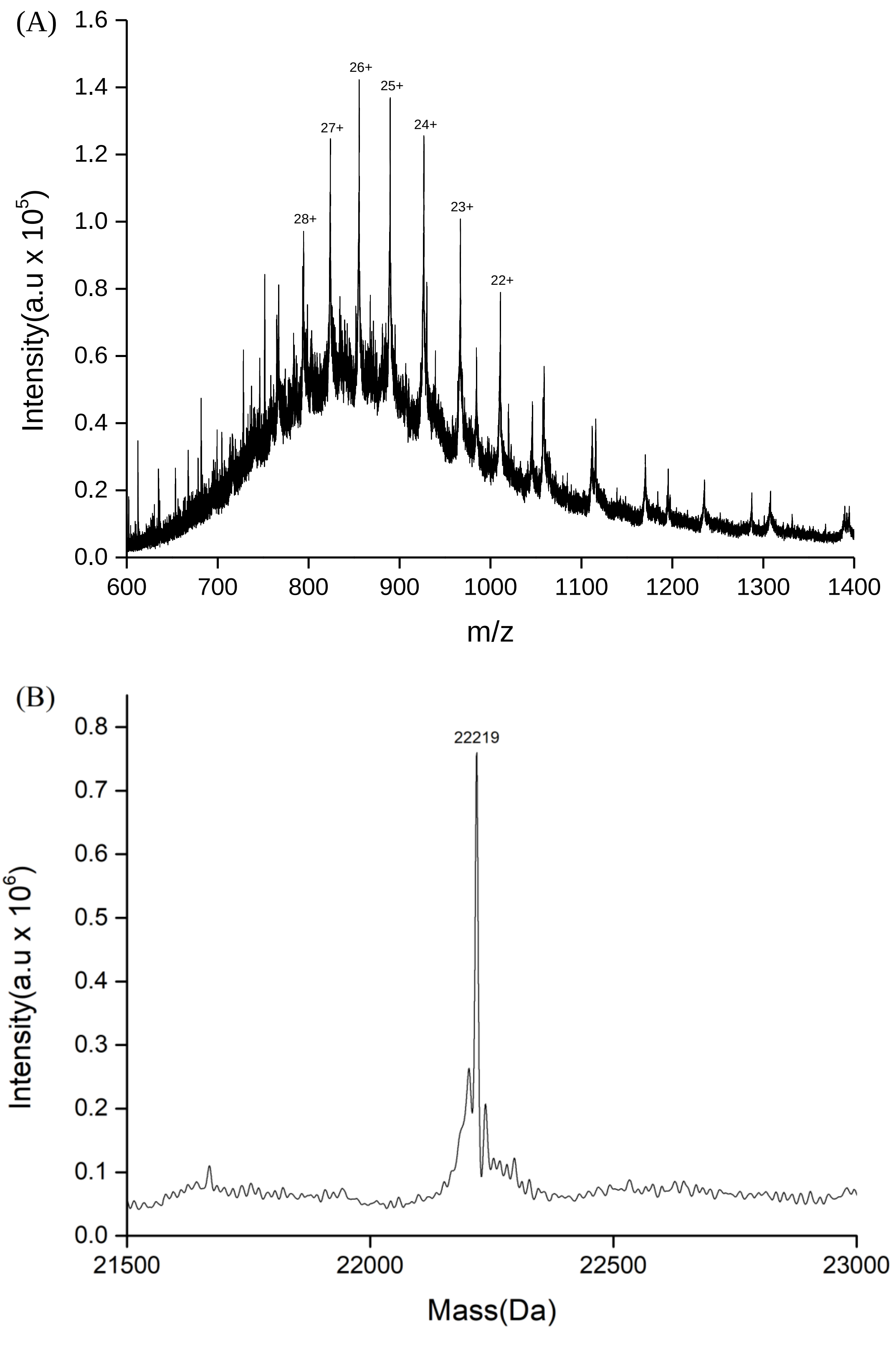

Figure S13. Molecular mass determination of Ftn-143-4.
(A) The ESI-MS and (B) the deconvoluted spectra of Ftn-143-**4**. Full-length Ftn-143-**4** is produced using AzFRS-MS•tRNA^{Pyl} pair in *E. coli* BL21 (DE3) supplemented with 1 mM IPTG and 1 mM **4** in GMML medium. The calculated molecular masse is 22,199 Da; observed molecular masses are 22,199 Da.

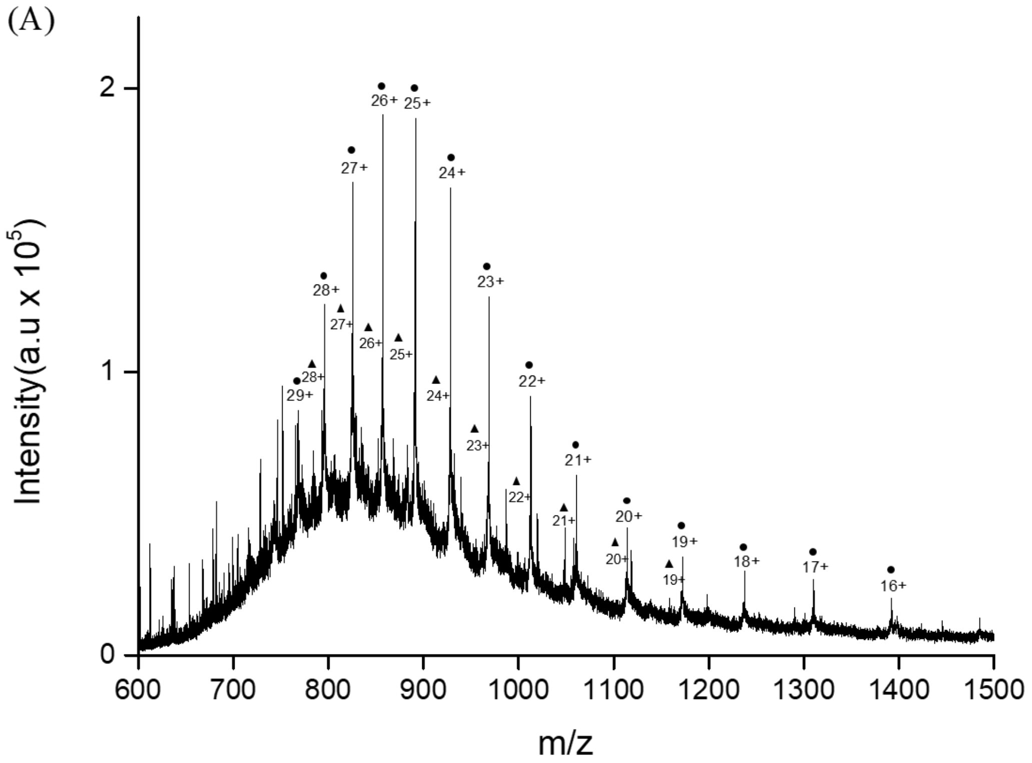

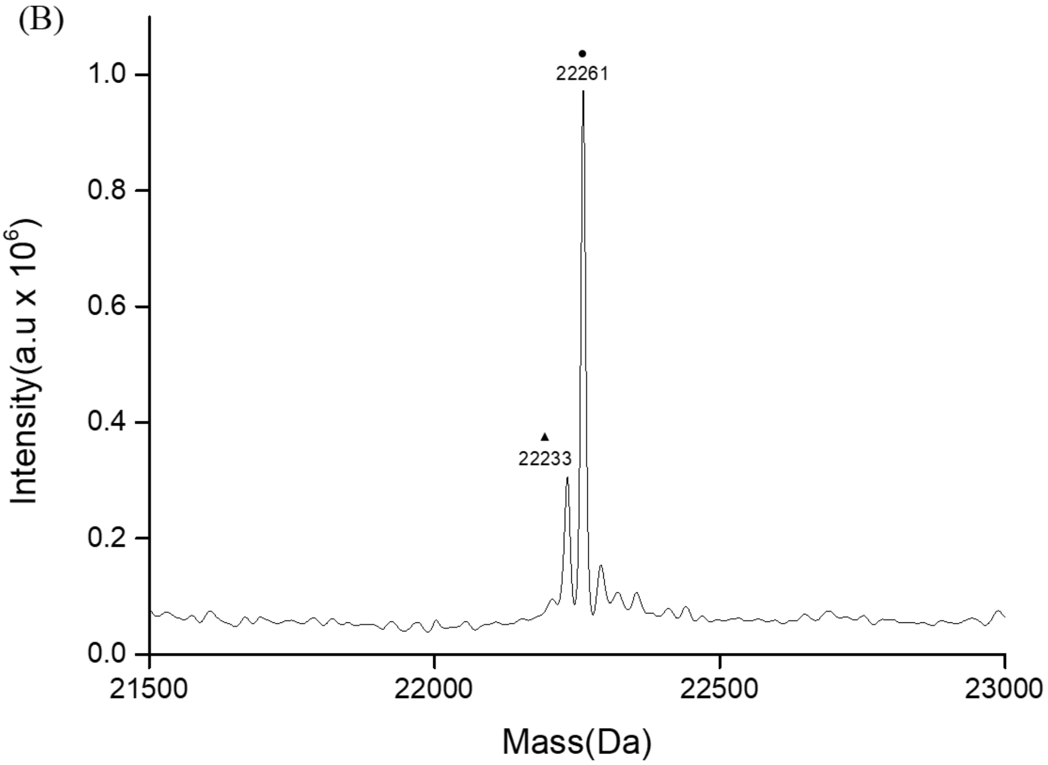

Figure S14. Molecular mass determination of Ftn-2am-4.
(A) The ESI-MS and (B) the deconvoluted spectra of Ftn-2am-**4**. Full-length Ftn-2am-**4** is produced using AzFRS-MS•tRNA^{Pyl} pair in *E. coli* BL21 (DE3) supplemented with 1 mM IPTG and 1 mM **4** in GMML medium. The calculated molecular masse is 22,261 Da; observed molecular masses are 22,261 Da and 22,233 Da (-N_3 converted to -NH_2 group).

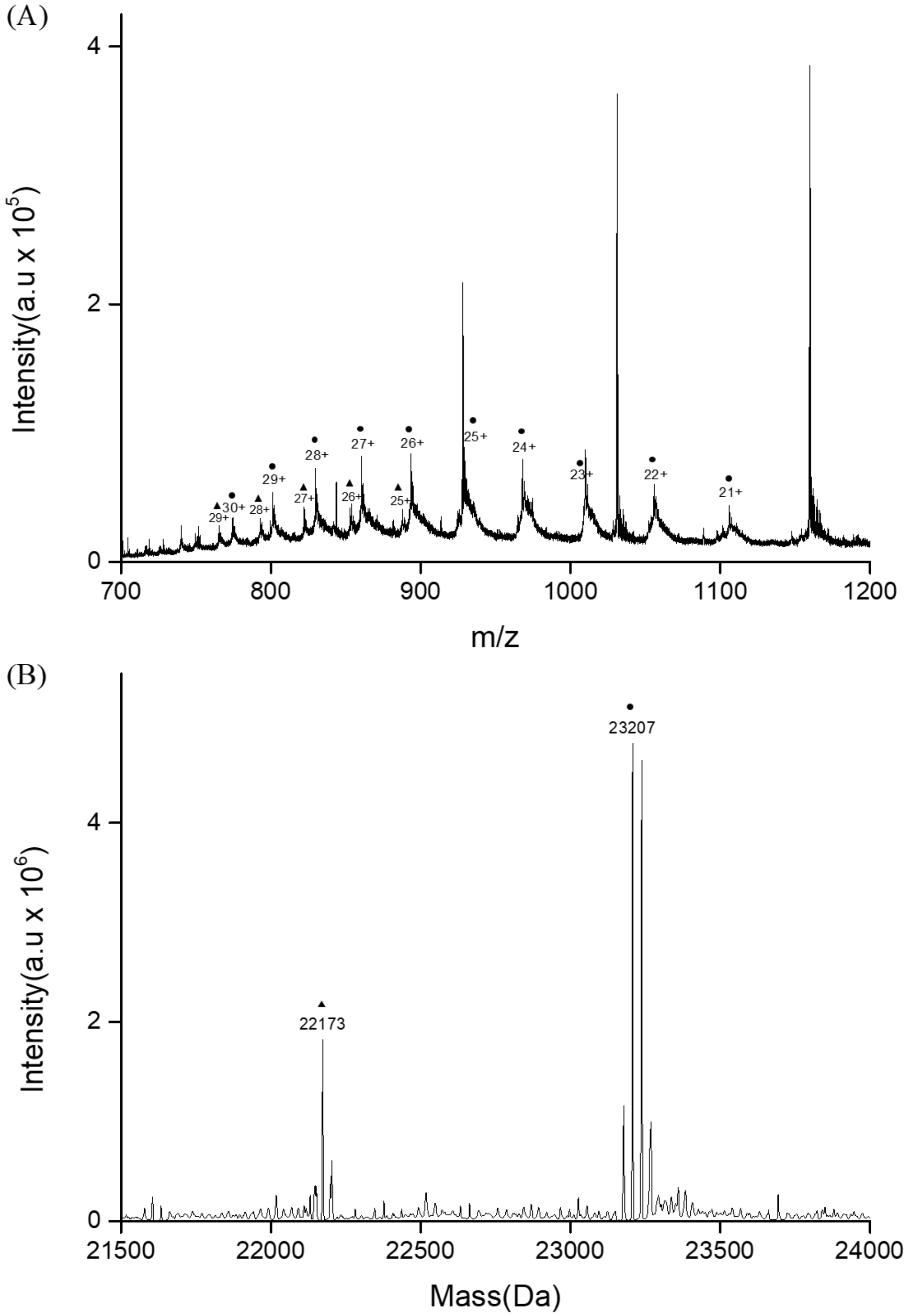

Figure S15. Molecular mass determination of Ftn-81-Cy5.
(A) The ESI-MS and (B) the deconvoluted spectra of Ftn-81-Cy5. Full-length Ftn-81- Cy5 is produced using AzFRS-MS•tRNA^{Pyl} pair in *E. coli* BL21 (DE3) supplemented with 1 mM IPTG and 1 mM **4** in GMML medium. The calculated molecular masse is 23,207 Da; observed molecular masses are 23,207 Da (one oxygen adduct also shown in the adjunct peaks) and 22,173 Da (-N_3 converted to -NH_2 group).

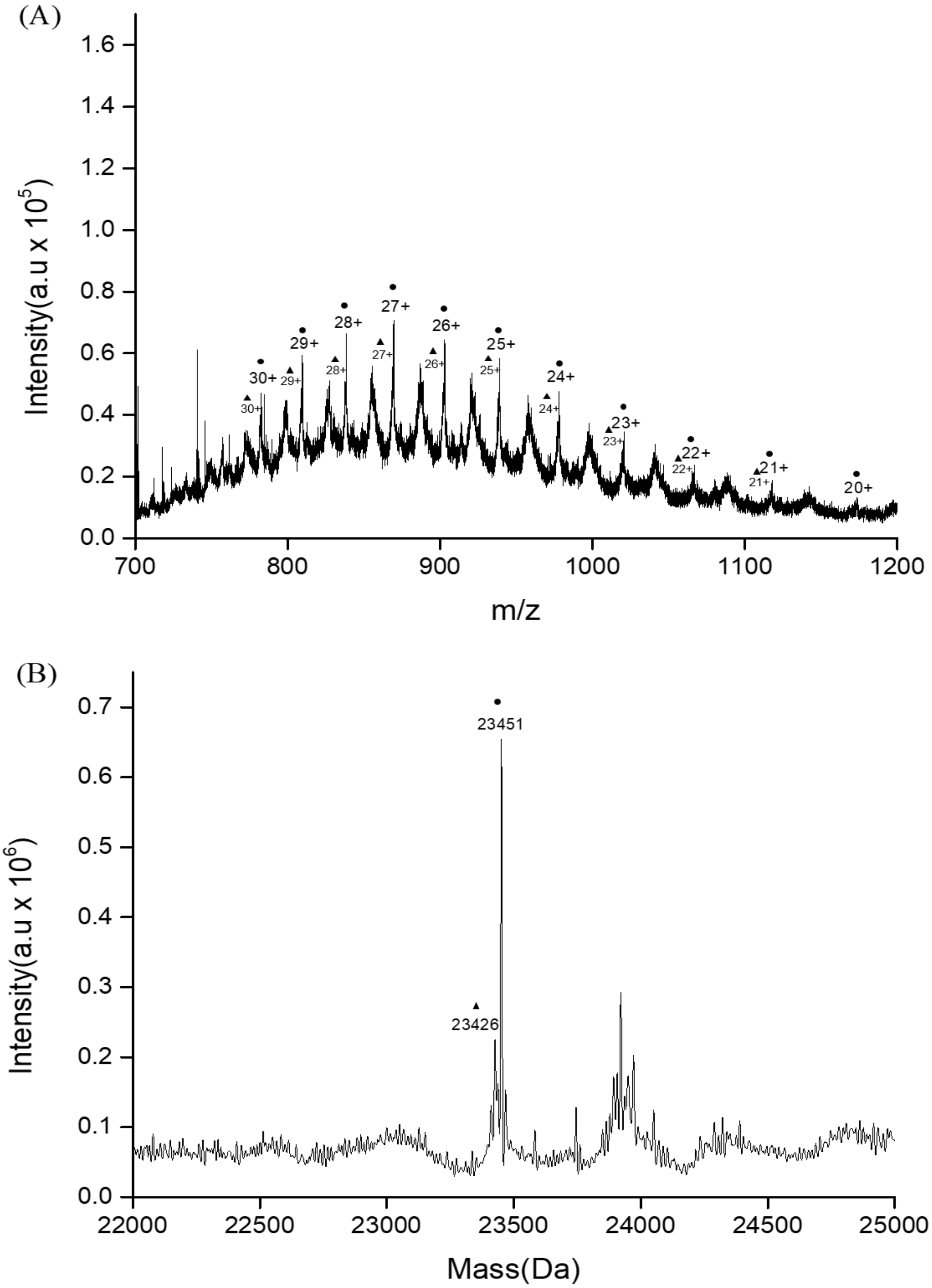

Figure S16. Molecular mass determination of A-Ftn-81-4.
(A) The ESI-MS and (B) the deconvoluted spectra of A-Ftn-81-**4**. Full-length A-Ftn-81-**4** is produced using AzFRS-MS•tRNA[Pyl] pair in *E. coli* BL21 (DE3) supplemented with 1 mM IPTG and 1 mM **4** in GMML medium. The calculated molecular masse is 23,448 Da; observed molecular masses are 23,451 Da and 23,426 Da (-N_3 converted to -NH_2 group).

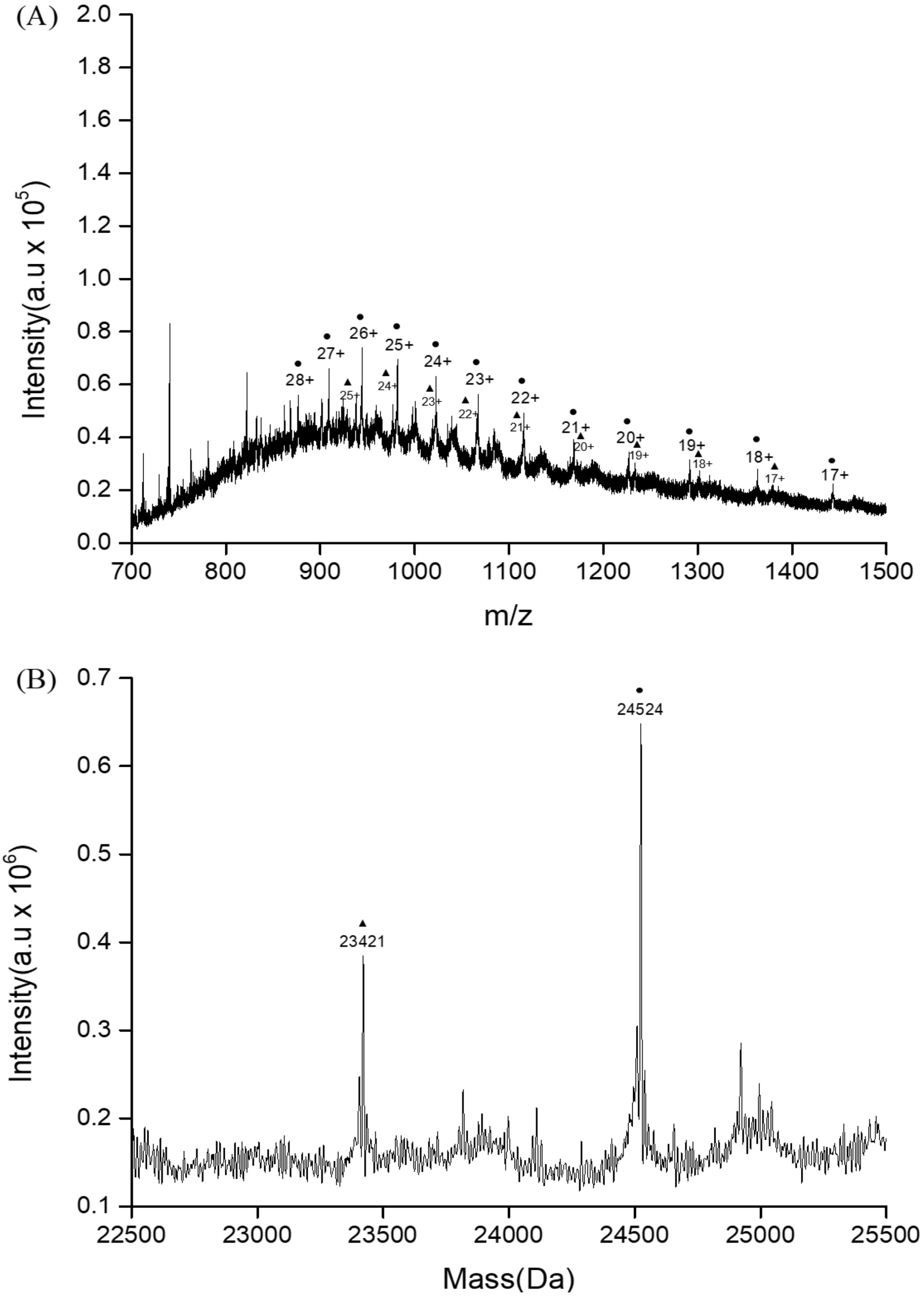

Figure S17. Molecular mass determination of A-Ftn-81-DOX.

(A) The ESI-MS and (B) the deconvoluted spectra of A-Ftn-81-DOX. Full-length A-Ftn-81-DOX is produced using AzFRS-MS•tRNA^{Pyl} pair in *E. coli* BL21 (DE3) supplemented with 1 mM IPTG and 1 mM **4** in GMML medium. The calculated molecular masse is 24,525 Da; observed molecular masses are 24,524 Da and 23,421 Da (-N_3 converted to -NH_2 group).

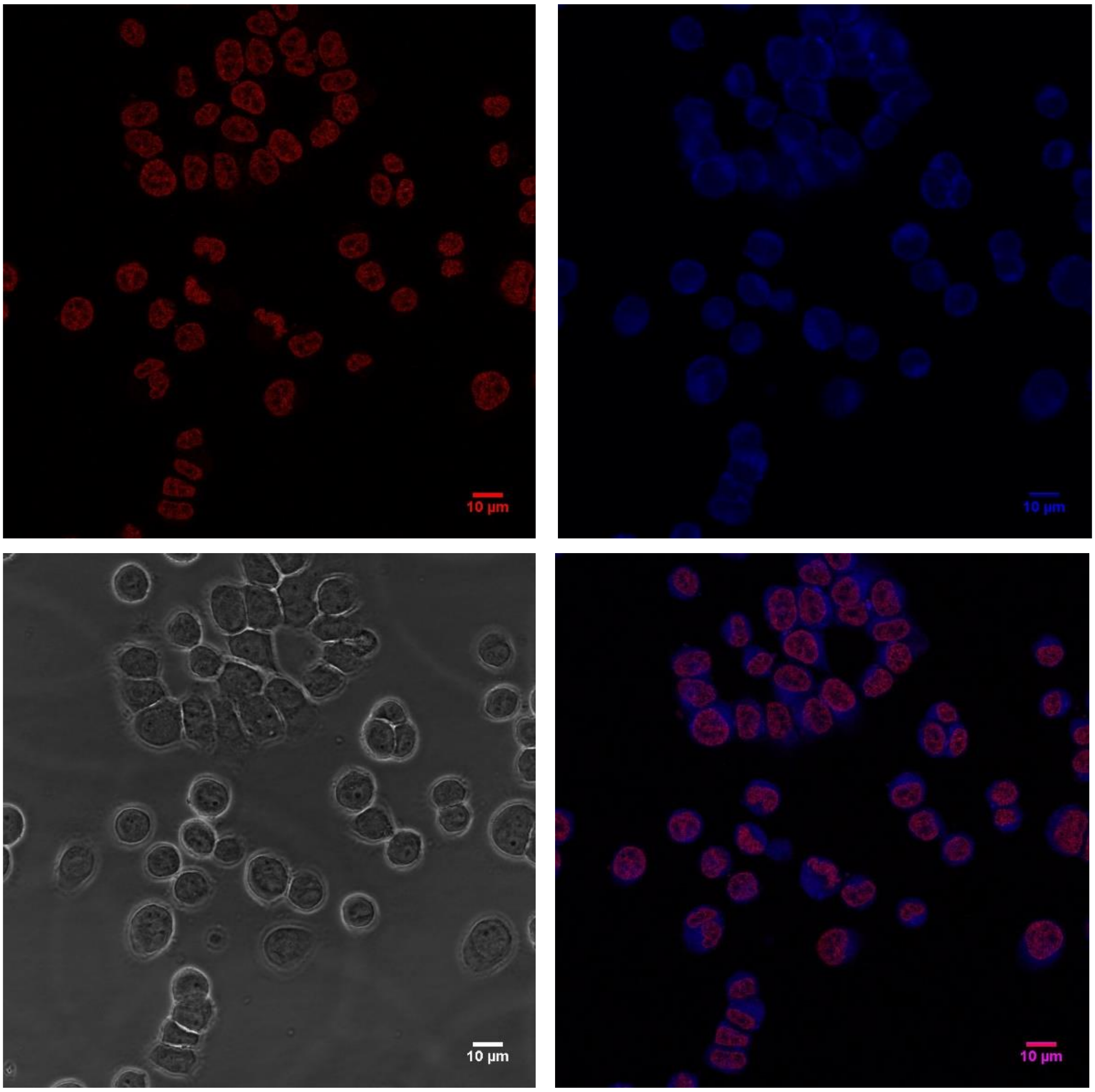

DOX	DAPI
Bright-field	Merge

Figure S18. Confocal microscopy fluorescence image analysis of BT474 cells incubating with 2 μM A-Ftn-143-DOX treating for 36 hours.

The cell nuclei are visualized by DAPI staining (blue); and by gel fluorescence emission image through DOX ($\lambda_{ex}/\lambda_{em}$ = 480 nm/590 nm, red).
